# FL496, an FL118-derived small molecule, induces growth inhibition, senescence, and apoptosis of malignant pleural mesothelioma (MPM) cells, and exhibits anti-MPM tumor efficacy strikingly superior to the pemetrexed-cisplatin combination

**DOI:** 10.1186/s13046-025-03547-9

**Published:** 2025-10-21

**Authors:** Ieman A. M. Aljahdali, Xiang Ling, Wenjie Wu, Wenchao Wang, Dan Li, Renyuan Zhang, Emma Zhang, Aimee Stablewski, Rami Azrak, Qingyong Li, Fengzhi Li

**Affiliations:** 1 Department of Pharmacology & Therapeutics, Roswell Park Comprehensive Cancer Center, Elm and Carlton Streets, Buffalo, NY 14263 USA; 2 Department of Molecular and Cellular Biology, Roswell Park Comprehensive Cancer Center, Buffalo, NY 14263 USA; 3 Canget BioTekpharma LLC, Buffalo, NY 14203 USA; 4 College of Pharmaceutical Sciences & Collaborative Innovation Center of Yangtze River Delta Region Green Pharmaceuticals & Key Laboratory of Marine Fishery Resources Exploitment & Utilization of Zhejiang Province, Zhejiang University of Technology, Hangzhou, 310014 China; 5 Department of Cancer Genetics & Genomics, Roswell Park Comprehensive Cancer Center, Buffalo, NY 14263 USA; 6 International Collaborations, Roswell Park Comprehensive Cancer Center, Buffalo, NY 14263 USA; 7 Developmental Therapeutics (DT) Program, Roswell Park Comprehensive Cancer Center, Buffalo, NY 14263 USA; 8 Present Address: College of Applied Medical Sciences, Department of Clinical Laboratory Sciences, Taif University, B.O. Box 11099, Al-Taif, 21944 Kingdom of Saudi Arabia

**Keywords:** FL496, FL118, Malignant pleural mesothelioma (MPM), P53, Mcl-1, Survivin/BIRC5, Bcl-2, DDX5, MPM tumor animal model

## Abstract

**Background:**

Malignant pleural mesothelioma (MPM) responds poorly to chemotherapy and is a highly progressive malignancy with a median survival time of only 6–9 months. Therefore, the development of anti-MPM tumor agents with high efficacy and low toxicity is urgent and addresses an unmet need for MPM patients.

**Methods:**

Medicinal chemistry synthesis of small molecules based on the FL118 drug platform were further comparatively investigated using multiple MPM and osteosarcoma cell/tumor in vitro and/or in vivo models. The method includes cell viability assay, Western blot analysis, colony formation assay, immunocytochemical staining, β-galactosidase senescence staining, flow cytometry, DNA fragmentation cell death detection, vector-free CRISPR-Cas9-mediated gene knockout, bioinformatic analysis, FL496 efficacy determination using severe combined immunodeficiency (SCID) mice with human MPM tumor, and immunohistochemistry (IHC) analysis of MPM tumors.

**Results:**

Here, we report that we identified a novel FL118-derived small molecule (FL496). FL496 appears to be strikingly more effective in inhibiting MPM tumor growth in MPM tumor animal models than the currently most prevalent pemetrexed-cisplatin combination in the clinic. The treatment of MPM cells with FL496 rapidly induced p53 and p21 accumulation, and Rb and p-Rb inhibition, which were associated with MPM cell senescence and G_1_/G_0_ arrest and apoptosis. Knockout (KO) of the TP53/p53 gene decreased the ability of FL496 to inhibit MPM cell growth (i.e., increase FL496 IC_50_ values) and colony formation. FL496-treated MPM cells resulted in strong inhibition of the expression of survivin, Mcl-1, Bcl-2, Bcl-XL, and the induction of active caspase-3, cleaved PARP, and PUMA, which were further confirmed using MPM tumor tissues via IHC analysis. High survivin in MPM patients’ tumors is associated with poor patient survival. Similar to FL118, FL496 treatment reduces DDX5 expression in MPM cells, but FL496 is more potent than FL118 in inhibiting MPM cell growth. Therefore, the mechanism of action (MOA) of FL496 overlaps with, but is likely beyond the scope of FL118 MOA, which needs further investigation.

**Conclusions:**

Together, these results indicate that FL496 is a promising anti-MPM small molecule, and its high anti-MPM potential is worthy of being further explored as a monotherapeutic agent to treat MPM patients in clinical trials.

**Supplementary Information:**

The online version contains supplementary material available at 10.1186/s13046-025-03547-9.

## Background

Malignant mesothelioma is a rare and aggressive cancer that develops from the mesothelial cells lining the lung (pleura), heart (pericardium), abdomen/belly (peritoneum), and testes (tunica vaginalis) [1]. To date, therapeutic trials have concentrated on malignant pleural mesothelioma (MPM), which forms in the lining of the lungs and accounts for 90% of diagnosed cases [1]. The 5-year survival rate of MPM patients is between 5% and 10% [2] and recent clinical trials indicated an overall survival of 14.2 months [3]. MPM’s causation relationship with asbestos and long fiber exposure is well-known and documented [4] however, the causality of other risk factors, such as exposure to the SV40 virus and radiation, is still disputed. In the United States, a partial ban on asbestos use was implemented in 1989, with a full ban on its use officially finalized in 2024 (US EPA Actions to Protect the Public from Exposure to Asbestos). While this ban will gradually decrease the incidence of MPM, the disease remains a global burden in the western world due in part to its long latency period (20–50 years), diagnostic difficulty, and limited treatment options for patients with advanced stages [1, 5].

In general, mesothelioma can be categorized into two histological categories: epithelioid, which accounts for up to 60% of recorded instances, and non-epithelioid, which includes the sarcomatoid (20%) and biphasic (20%) subtypes. Survival is closely associated with histological subtypes in most patient outcomes. Patients with epithelioid histology have a 12-17-month life expectancy; patients with sarcomatoid histology have 7-18-month life expectancy; and patients with biphasic tumors have 8-21-month life expectancy, highlighting the effect of histological categorization on mesothelioma patient outcomes [5–8].

A novel small-molecule compound named FL118 was identified by high-throughput screening using the inhibitor of apoptosis (IAP) protein survivin (i.e., BIRC5/baculoviral IAP repeat containing 5) gene promoter-driven luciferase reporter as a biomarker and target in cancer cells [9]. Studies over the past decade have shown that FL118 is a highly effective anticancer agent for treating a variety of human cancers, including head-and-neck and colorectal cancers [9–14], multiple myeloma [15–17], pancreatic cancer [14, 18, 19], prostate cancer, and osteosarcoma [19]. Furthermore, FL118 serves as an excellent drug platform for the development of a wide range of novel FL118 analogues [20, 21]. We provided evidence in a review article showing that an FL118 derivative (FL496) appears to have a unique (but overlapped with FL118) antitumor mechanism of action (MOA) for a rare kidney cancer with genetic defects in fumarate hydratase (FH) production [22], which is classified as FH deficiency-induced type-2 papillary renal cell carcinoma (FHpRCC) [23]. Another FL118-derived compound (FL7N1) appears to be suitable for treating retinoblastoma [24], a rare pediatric cancer. Additionally, we have obtained yet-to-be published data showing that FL496 also demonstrated excellent anti-tumor activity against retinoblastoma – a rare cancer with a distinct tumor origin and oncogenic drivers compared to rare kidney cancer. Given the current therapeutic options for MPM, while these observations do not gurantee that FL496 will be a good treatment for MPM, they provide reasonable justification to further explore the concept, especially since MPM is very aggressive, presently lacks an effective treatment option and is in desperate need of novel treatments. Therefore, we chose MPM, a rare cancer, as a model system to evaluate the potential of FL118, FL7N1 and FL496 in the ability of reducing mesothelioma cell viability. Our findings indicate that FL496 is a significantly superior candidate among these small molecules.

In the study reported here, we first performed an in vivo screening of three heterocyclic ring compounds (FL7N1, FL7N2, FL7N3) to identify the best of the three compounds with heterocyclic ring, followed by the in vitro IC50 comparison of the identified candidte (FL7N-1) with FL118 and FL496. We found that FL496 is the best candidate among FL7N1, FL118 and FL496 in terms of the inhibtion of MPM cell growth/viability. We then focused on the in vitro and in vivo studies of FL496 using MPM cancer models plus osteosarcoma models. Our results suggest that FL496 holds strong potential for further development toward clinical trials as a montotherapy agent for treating MPM patients.

## Methods

### Synthesis of FL118 and FL118 analog compounds relevant to this study

**Scheme 1:**
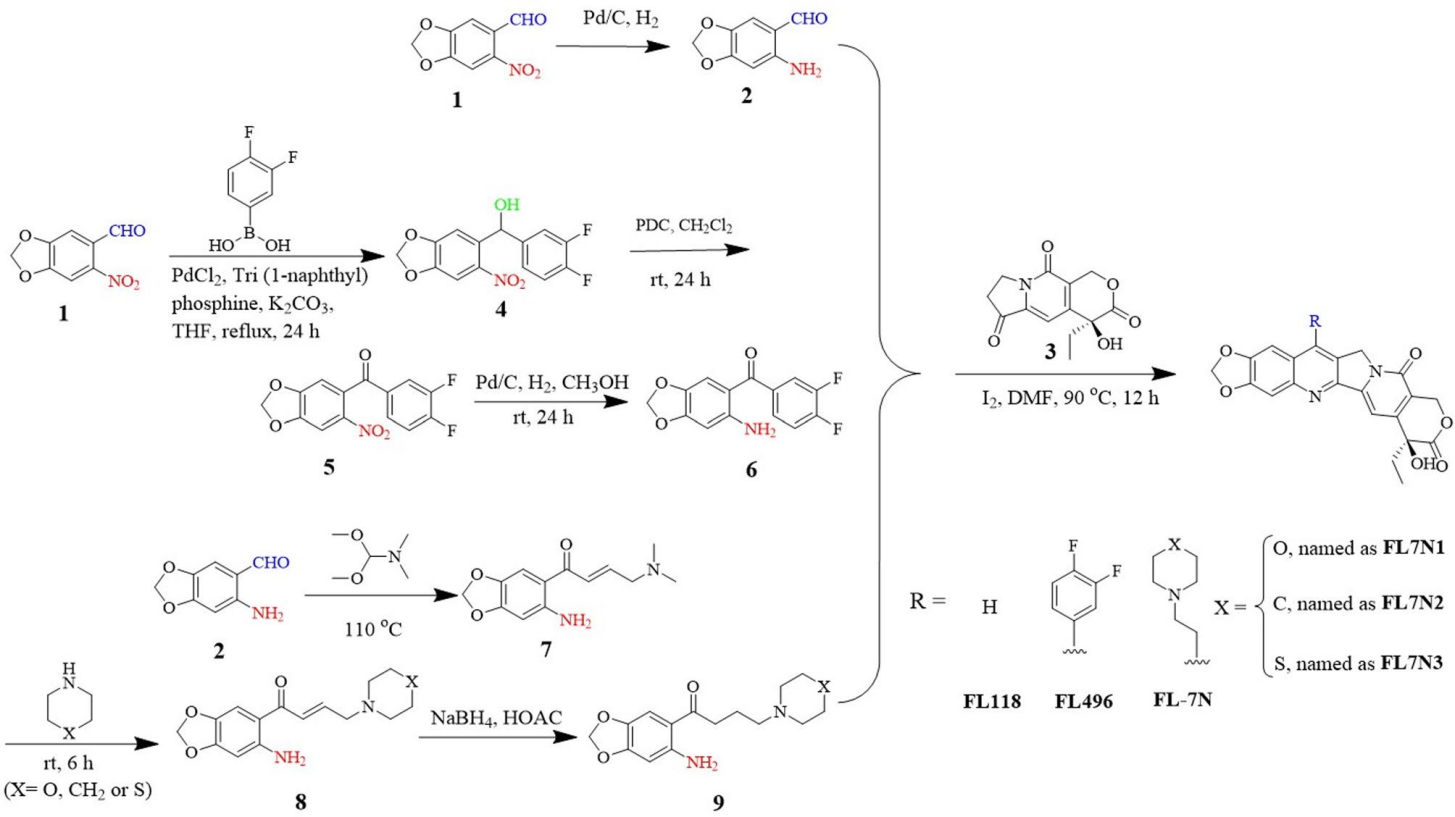
Outline the **synthesis of FL118 and FL118 analogs that are relevant to this study**

We described the synthesis of various FL118-derived analogs that are relevant to this project in detail in our previous studies [20, 24]. Briefly, FL118 was synthesized as follows (Scheme 1): 6-Nitropiperonal (1) was reduced with Pd/C in methanol under H_2_ for 12 h, obtaining intermediate (2) with 90% yield. Stirring intermediate (2) and (4 S)-4-ethyl-7,8-dihydro-4-hydroxy-1 H-pyrano[3,4-f]indolizine-3,6,10(4 H)-trione (3) at 90 °C in DMF in the presence of iodine, obtaining FL118 with 65% yields.

FL496 (Hx-6) was synthesized as follows (Scheme 1): 6-Nitropiperonal (1) was coupled with 3,4-Difluorophenylboronic acid using PdCl_2_, Tri(1-naphthyl)phosphine, and K_2_CO_3_ in THF at 65 °C for 24 h, to form intermediate (4) with 68% yield. Intermediate (4) was oxidized with pyridinium dichromate (PDC) in CH_2_Cl_2_ at room temperature (rt) for 24 h, obtaining intermediate (5) with 85% yield. Intermediate (5) was reduced using Pd/C under H_2_ in methanol for 12 h, obtaining intermediate (6) with 90% yield. Compounds (3) and intermediate (6) were stirred at 90 °C in DMF with iodine, obtaining FL496 with 65% yield.

FL7N compound series were synthesized as follows (Scheme 1): Intermediate (2) was reacted with DMF at 110 °C for 2 h. After cooling to room temperature (rt), hexane was added, and the mixture was filtered to obtain a yellow solid intermediate (7) with 95% yield. Intermediate (7) was dissolved in dioxane, treated with morpholine, hexahydropyridine, or thiomorpholine, respectively, and then heated under reflux for 6 h to produce intermediate (8) with 80% yields. Intermediate (8) was dissolved in glacial acetic acid, and sodium borohydride (NaBH_4_) was added at 0 °C. The reaction was stirred for 3 h to produce intermediate (9) with 70% yield. Compounds (3) and intermediate (9) were stirred with iodine in DMF at 90 °C, obtaining FL7N with 10–15% yield.

These products are characterized by ¹H NMR and ¹³C NMR. FL496: yellow solid; m.p.>250 °C; ^1^H NMR (500 MHz, CDCl3) δ 8.04 (s, 1H), 7.78 (s, 1H), 7.55 (q, J = 8.4 Hz, 1H), 7.33 (dd, J = 16.2, 7.8 Hz, 1H), 7.23 (s, 1H), 7.19 (s, 1H), 6.39 (s, 2 H), 5.70 (d, J = 16.9 Hz, 1H), 5.39–5.25 (m, 3 H), 1.90 (q, J = 7.4 Hz, 2 H), 1.00 (t, J = 7.3 Hz, 3 H). ^13^C NMR (126 MHz, TFA-sxhuo) δ 175.62, 157.35, 152.58, 139.88, 139.11, 139.06, 128.28, 127.53, 127.17, 124.17, 121.40, 118.73, 118.58, 116.90, 115.24, 114.41, 112.99, 112.16, 104.60, 103.56, 101.32, 96.78, 73.22, 65.65, 50.88, 30.48, 5.19. HR-MS calcd for C27H18F2N2O6 [M + H]+: 505.1211; found 505.1232; [M + Na]+: 527.1031; found 527.1056. Others were reported previously [24].

### Research reagents and antibodies

SN-38 (active irinotecan metabolite) was purchased from Selleckchem (Houston, TX, USA). Pemetrexed disodium (HY-10820 A) was purchased from MedChemExpress (MCE, Monmouth Junction, NJ, USA). Cisplatin (232120) was purchased from EMD Millipore Corp. (St. Louis, MO, USA). Etoposide was purchased from Sigma-Aldrich (St. Louis, MO, USA). The synthesized FL118, FL7N1, FL7N2, FL7N3 and FL496 have a purity ≥ 99%. A list of antibodies used in this project is summarized in Supplemental Table S1.

### Preparation of relevant drugs for in vitro and in vivo studies

For in vitro studies, the powder compound of FL118, FL7N1, FL496, and SN38 was first dissolved in DMSO at 1 mM stock and stored in aliquots at -80 °C. These stock solutions were further diluted with ddH2O for in vitro or cell culture media (ex vivo) to the final concentrations needed for the planned studies. For in vivo studies, FL118, FL7N1, or FL496 was formulated with the ethanol-dissolved HPβCD (2-hydroxypropyl-β-cyclodextrin) solution, followed by lyophilization to obtain a HPβCD-drug complex powder without ethanol. FL496 (i.e., HPβCD-FL496 complex), pemetrexed and cisplatin were prepared using normal saline to a final concentration that is convenient for dosing in animals at the dose level of 1.25–7.5 mg/kg (FL496), 15–30 mg/kg (pemetrexed), and 1.5-3 mg/kg (cisplatin), respectively.

### Cell lines and cell culture

Human MPM cancer cell lines, MSTO-211H and NCI-H226 have been used in numerous publictions, including recent ones [25–27]. We purchased MSTO-211H and NCI-H226 from ATCC. MSTO-211H and NCI-H226 were maintained in RPMI-1640. The human osteosarcoma cell line U2OS and human cervical cancer cell line HT-3 were was purchased from ATCC and maintained in DMEM media. Pediatric osteosarcoma patient-derived xengraft (PDX) tumor cell lines, OS152, OS742, OS766 and OS833 were gifts from either Dr. Alejandro Sweet-Cordero Lab at UCSF [28] or getting his permission to obtain them from other lab. These PDX cell lines were cultured in DMEM. All culture media were supplemented with Fetal Bovine Serum/FBS (Atlanta Biologicals^™^ or Invitrogen/Thermo Fisher Scientific, USA) to a final concentration of 10% together with 1% of Penicillin/Streptomycin (CORNING, Manassas, VA, USA) at 37 °C in a 5% CO_2_ humidified incubator as a monolayer culture. Cells were harvested with 0.25% trypsin (CORNING, Manassas, VA, USA) and were passaged twice per week for maintenance.

### Cell viability (MTT) assay

The principle was described in our previous studies [9] and the MTT method was slightly modified in this study. Briefly, for cell viability measurement, 24 h before treatment, 4 × 10^3^ cells per well in 100 µl culture media were seeded in 96-well plates. On the following day, cells were treated with 100 µl of the planned therapeutic drug(s) diluted in cell culture media using a manual serial dilution method or the HP D300 Digital Drug dispenser (Tecan, Durham, NC, USA). Plates containing the treated cells were placed back in the incubator for 72 h. In 96 well plates, cell viability was measured by adding 40 µl of 0.15 mg/ml Resazurin Sodium salt in 1X DPBS (Sigma, Cat# R7017) to each well and incubating for up to 4 h. Fluorescence was monitored with a plate reader (Synergy2 plate reader by BioTek) using the 560 nm excitation/ 590 nm emission filter set. The dose-response data were processed with Excel or GraphPad to calculate the EC50/IC50.

### Immunoblotting /western blot analysis

Following various treatments, cells in cell culture plates were washed with ice-cold 1X PBS before harvesting cells using 0.025% Trypsin. Cells were then collected into 15 ml tubes and pelleted by centrifugation at 1,000 rpm at 4 °C for 3–5 min. After washing the cell pellet once with ice-cold 1X PBS and centrifuging again at 1,000 rpm at 4 °C for 3–5 min, cell pellets were lysed using 1X SDS lysis buffer prepared from 10X cell lysis buffer (Cell Signaling, Cat# 9803) using ddH_2_O and containing 1 mM PMSF (Sigma-ALDRICH) or complete EDTA-free protease inhibitor cocktail tablets (Roche, Cat#04693132001). Cell lysates were transferred to fresh, labeled Eppendorf tubes and kept on ice for 30 min with frequent vortexing before centrifugation at 13,000 rpm at 4 °C for 10 min. Supernatants were then used for protein concentration determination using the Pierce BCA assay kit (Thermo Scientific, Cat# 23225) following the manufacturer’s instructions. Then 30–50 µg of protein per sample/well was separated on SDS-PAGE gels in parallel with BLUeye prestained protein ladder (GeneDirex, Taiwan) using MINI-PROTEIN TGX, 4–15% (Bio-Rad, Cat#456083) with 1X Tris/Glycine/SDS Buffer (Bio-Rad, Cat#1610732) according to manufacturer’s recommendations. Gels were then transferred to 0.45-µm Nitrocellulose membranes (Bio-Rad,162 − 0115) for 1 h in cold 1X Tris/Glycine buffer (Bio-Rad, Cat#1610734 + 20% methanol) using a transfer apparatus at a voltage setting of 100 V. Membranes were then blocked in 5% milk in TBS-T buffer for 2 h at room temperature and then incubated with primary antibodies (Supplemental Table S1) prepared in 3% Bovine Serum Albumin (BSA) in TBS-T overnight at 4 °C. After three consecutive washes of 10 min with 1X TBS-T, membranes were incubated with secondary antibodies in 5% milk in 1X TBS-T for 2 h at room temperature. Membranes were then washed three times for 15 min with 1X TBS-T. After washes, Western blot signal detection was performed using Clarity™ Western ECL substrate-Luminol Solution (Bio-Rad, Cat# 1705602 S) and exposed to X-Ray films (FUJIFILM, Japan) for various times (5 s to 30 s) to visualize the result.

### Colony-forming assay

A 2-ml media with 50–100 cells was seeded in each well of 12-well plates. After 24 h of attachment, cells were treated with a drug or vehicle for 2 h, 24 h, and 72 h, respectively, before being replaced with drug-free media. Cells were then allowed to grow for two weeks in the incubator with media changed every 72 h to allow the cell colonies growing in sufficient size (> 50 cells). Then, the media was aspirated, and wells containing colonies were washed with 1X PBS and fixed with 1 ml of 100% methanol for 5–10 min. Methanol was then aspirated, and colonies were stained using 1 ml of 0.5% Crystal violet for 20 min. Plates were then gently washed with tap water until the dye cleared and allowed to dry before counting using ImageJ for quantification. Pictures for cell colonies in the 12-well plates were taken using a Nikon D3300.

### γH2AX foci assay using γH2AX immunocytochemical staining

Gaudreau-Lapierre, A. et al.’s method [29] was adapted and applied to our studies. Briefly, MPM cells were grown on 2% gelatin (Sigma, MO)-coated 12 mm micro circle cover glass (VWR, PA) in 12-well cell culture plates overnight in the incubator at 37 °C with 5% CO_2_. The next day, cells were treated with either 20 nM FL496 or 20 µM Etoposide for 0, 0.5, 1, 2, 4, and 6 h. At the time of collection, media was aspirated from wells containing slides and washed twice with ice-cold immunofluorescence (IF) washing solution (1X PBS containing 0.05% Tween-20) while on ice. Cells were then fixed with a fixative solution (3% Paraformaldehyde + 2% sucrose in 1X PBS) on ice for 5 min and then permeabilized for 15 min at room temperature before washing twice with IF washing buffer. Coverslips were then blocked with blocking buffer (1X PBS containing 3% BSA and 0.05% Tween-20) for 30 min at room temperature. Coverslips were then incubated with primary antibodies (p-H2AX ser139, Millipore, Cat#05-636) that were diluted in blocking buffer overnight at 4 °C. On the second day, cells were washed four times for 5 min each with IF washing buffer before incubating them with secondary FITC-labeled antibodies (Invitrogen, Cat#62-6511) diluted in blocking buffer for one hour at 37 °C protected from light. Slides were then washed with IF washing buffer and incubated with one µg/µl DAPI (Roche, Cat#10236276001) in 1X PBS for 5 min. Coverslips were picked using forceps and placed on microscopic slides (Fischer brand, 12-550-14) using 8–10 µl of mounting media. Slides were kept dark and taken for visualization using an IF microscope (OLYMPUS, IX73 microscope system). Images were then captured using the cellSens software as TIF files.

### Senescence-associated β-galactosidase staining

A β-galactosidase staining assay was performed following the manufacturer’s instructions using the histochemical staining for senescence cells kit (Sigma-Aldrich, Cat# CS0030). Briefly, 100–200 cells in 2 mL per well were plated on 12-well plates and allowed to attach overnight. Cells were then treated with a drug or vehicle for 2 h, 24 h, and 72 h, followed by medium replacement with drug/vehicle-free media. Cells in the plates were continuously cultured in an incubator for four days. After the cells were carefully washed twice with 1 mL of 1X PBS per well without cell detachment, 1.5 mL of 1X fixation buffer was added to each well and incubated for 6–7 min at room temperature. Cells were then rinsed three times with 1 mL of 1X PBS before being stained with 1 mL of staining mixture per well. Plates were then kept in a 37 °C incubator without CO_2_ overnight. The next day, cells were observed under the microscope, and the number of greenish/blue-stained cells was counted among the total number of cells. The percentage of cells expressing β-galactosidase positivity (senescent cells) of the total cell population for a given treatment group was plotted using Microsoft Excel. Pictures for cell senescence were taken under a Olympus Fluorescence Microscope with an overheal camera.

### Flow cytometry analysis of cell cycle distribution

Cells in 6-well plates were treated with a vehicle or FL496 at a concentration of 10 nM and 100 nM for 24 h and 48 h after synchronization by serum starvation overnight. Cells were then harvested by trypsinization and resuspended in 1 ml 1X PBS. Cells from each well were then fixed for two hours by dropwise adding 0.7 ml 70% ice-cold ethanol during gentle agitation. The fixed cells were resuspended in staining buffer (1X PBS with 100 µg/ml RNase A, 50 µg/ml Propidium Iodide, and 0.1% Triton X-100) and wrapped in foil for light protection before analysis. Cells were analyzed within 1–2 h on a FACSCalibur™ flow cytometer (BD Biosciences, San Jose, CA, USA) using forward-scattered light versus side-scattered light as gating parameters. ModFit LT™ 5.0 (Verify Software House, Topsham, ME) or FCS Express 7 (De Novo Software, Pasadena, CA, USA) was utilized for gating and flow cytometric data.

### DNA fragmentation cell death detection

The experiment was performed following our previous method using a Roche Cell Death Detection ELISA^Plus^ assay kit [30]. Briefly, after MPM cells were seeded into 48-well plates (3 × 10^4^ cells per well) overnight, cells were then treated with vehicle (DMSO) and FL496 at 10 nM and 100 nM for 24 h and 48 h, respectively. After medium in each well was removed and cells in each well were gently washed with room temperature 1X PBS one time, cells in the 48-well plates were lysed with 200 µl lysis buffers (in the kit) per well for 30-min incubation at room temperature. Then, cell lysates in the plate were centrifuged at 200*g* for 10 min. Twenty microliters of aliquots from the supernatant were dispensed into streptavidin-coated 96-well plates followed by the addition of 80 µl of immuno-reagents. The immuno-reagent consisted of a mixture of anti-histone biotin and anti-DNA-HRP directed against various histones and antibodies to both single-stranded DNA and dsDNA, which are major constituents of the nucleosomes. After 2 h incubation at room temperature with gentle shaking, unbound components were removed by washing with 250 µl of 1X incubation buffer. One hundred microliters of 1X HRP substrate (2,2′-azino-di-(3-ethylbenzthiazoline-6-sulfonnic acid) diammonium salt in ABTS solution was added to each well, and the plate was then put on a shaker at 250 rpm for color development. Measurements were made at 405 nm against an ABTS solution as a blank (reference wavelength ∼ 490 nm) using an Ultra Microplate Reader (Bio-Tek Instruments).

### Knockout (KO) of TP53 in MPM cells using vector-free CRISPR-Cas9 technology

Genetic disruption of TP53 (p53 gene) in Mesothelioma cells was performed using vector-free CRISPR-Cas9 technology following the previously established procedure [14]. Briefly, two guide RNAs (sgRNAs) targeting TP53 exon 3 and exon 4 were ordered from Integrated DNA Technologies, Inc. (IDT). The TP53 sgRNA was mixed with Cas9 enzyme protein to form sgRNA-Cas9 ribonucleoprotein (RNP) complex, and then transfected into mesothelioma cells through electroporation for knockout (KO) of TP53 expression in mesothelioma cancer cells. To isolate clones with TP53 KO, the transfected cells were sorted by flow cytometry, and then we used a limited dilution of the flow cytometry-sorted positive cell pool in a series of 96-well plates to generate single-cell clones in individual wells of 96-well plates. Individual single cell clones were selected and expanded in 12-well plates. A portion of each close was processed for targeted sequencing of human TP53 exons 3 and 4, corresponding to the sgRNA-targeted deletion site. As a result, 10 clones from MSTO-211H and 26 clones from NCI-H226 were identified with $\:\ge\:$70% and $\:\:\ge\:$99% TP53 deletion, respectively (Supplmental Figures S1, S2; Supplemental Tables S2- S4). Meanwhile, we collected the PCR-confirmed wild typle p53 clones (Supplemental Figure S1A) and determined p53 protein expression from each single clone by Western blots. To further confirm the p53 KO cell clones, we performed immunoblotting for p53 and p21 after treating the cells with 20 µM etoposide for 24 h to induce DNA damage and activate p53 and p21 expression to verify parental MPM cells in response to DNA damage in p53 wild-type parental mesothelioma cells, which served as the control parental cells for comparison with the p53 KO cells. We finally isolated 3 cell clones from each mesothelioma cell line with TP53 KO. The TP53 KO cell clones from MSTO-211H cells were designated as “18 A,” “11 C,” and “18E”, and the TP53 KO cell clones from NCI-H226 cells were designated as “9E,” “4D,” and “3D” based on the well they originated from, and these names were used throughout the report.

### Bioinformatic analysis of survivin/BIRC5 expression with MPM patient survival association

RNA sequencing data on survivin/BIRC5 and clinical information from patients diagnosed with mesothelioma were obtained from the TCGA database. Specifically, we downloaded the publicly available GDC TCGA-MESO (*n* = 84 primary tumor) dataset from the UCSC Xena browser. Clinical information, survival status, and RNA-seq data were then combined. Normalized RNA-seq data were converted into transcripts per million (TPM) and then transformed to log2 (TPM + 1) for chart plotting using the R language (R version 4.0.3: www.r-project.org). A t-test was used to evaluate the statistical significance of the mRNA expression level in eMPM versus bMPM tumor tissues. Kaplan-Meier survival analyses of overall survival for TCGA-MESO patient cohorts were performed as follows: Based on the mRNA expression of the BIRC5/surviving gene, mesothelioma patients were grouped into the high survivin expression group or low survivin expression group. The *p*-value for the significance of high versus low BIRC5/survivin gene expression was calculated using the log-rank test. The figures were created using R version 4.0.3.

### Comparative antitumor efficacy studies using human tumor animal models

Animal care and experiments were performed by the animal protocol approved by the Institutional Animal Care and Use Committee (IACUC) at Roswell Park Comprehensive Cancer Center (Protocol number: 1192 M). The MPM MSTO-211H cell-established tumor was used as a tumor model in SCID mice to assess the efficacy of FL496. Briefly, xenograft tumors were first established from the MSTO-211H cell line by subcutaneously injecting 1 × 10^6^ cells at the flank area of SCID mice in 100 µl volumes mixed with Matrigel (Corning 354234) at a ratio of 1:1. The derived tumor was then passed 1–2 generations in SCID mice, and tumors were maintained on SCID mice. Experimental tumor mice were prepared by transplanting 30–50 mg of non-necrotic tumor mass using a trocar from tumor maintenance mice after tumors reached ~ 800–1000 mm^3^. Forty-five female SCID mice, ages 8–10 weeks (5 mice per group) bearing MSTO-211H xenograft tumors (with a size of 150–250 mm^3^) were randomly divided into nine groups at week 1. Based on the previously published studies for the use of pemetrexed and cisplatin combination treatment for preclinical animal model studies [31–34], the 9 SCID mouse groups bearing MSTO-211H xenograft tumors were then treated as follows: (1) saline (*vehicle control*); (2) pemetrexed 15 mg/kg + cisplatin 1.5 mg/kg (*15 pem + 1.5 Cis*); (3) pemetrexed 30 mg/kg + cisplatin 3 mg/kg (*30 pem + 3 Cis*); (4) pemetrexed 30 mg/kg + newly prepared cisplatin 3 mg/kg (*30 pem + 3 Cis New*); (5) FL496 5 mg/kg (*5 mg/kg FL496*); (6) newly prepared FL496 5 mg/kg (*5 mg/kg FL496 New*); (7) FL496 7.5 mg/kg (*7.5 mg/kg FL496*); (8) pemetrexed 15 mg/kg + cisplatin 1.5 mg/kg + FL496 1.25 mg/kg (*15 Pen + 1.5 Cis + 1.25 FL496*) and (9) pemetrexed 30 mg/kg + cisplatin 3 mg/kg + FL496 5 mg/kg (*30 pen + 3 Cis + 5 FL496*). The treatment schedule was weekly x 4 for the vehicle control and FL496. The schedule of pemetrexed was five times per week for two weeks, and cisplatin once weekly for two weeks. All drugs were administered via the intraperitoneal (ip) route. The data (tumor size, animal body weight, and other toxicity parameters) were documented over time. Experiments were maintained until all mice were dead (sacrificed due to a large tumor) or until 98 days (14 weeks) from the first treatment. Following IACUC guidelines, animals with a moribund state (judged by animal body weight loss, behavior, fur status, movement, and diarrhea) or tumor oversize (≤ 2000 mm^3^) were euthanized.

### MSTO-211H MPM tumor tissue collection, H&E staining and immunohistochemistry analyses

SCID mice (3 mice per group) bearing MSTO-211H tumor at 400–500 mm^3^ were then treated with vehicle or FL496 at 5 mg/kg (weekly x 4) alone or in combination with pemetrexed (30 mg/kg, 5 times a week for 2 weeks) + cisplatin ( 3 mg/kg, weekly x 2). Tumors were collected at 4 weeks (wks) and 8 wks, respectively. Collected tissues were immediately soaked in 10% NBF Zinc solution for 24 h and then transferred into 70% ethanol for 24 h to 72 h. During this period, tissues were processed to be embedded into paraffin block cassettes for cutting and analyses. Dako CoverStainer was utilized for H&E staining on tissue slide sections from the paraffin-embedded tumor tissues with a DAKO H&E kit. Immunohistochemistry (IHC) analyses of various tissue slide sections used corresponding antibodies for cleaved/active caspase-3, γH2AX, Ki67, survivin, and Bcl-xL. Tissue slide sections were prepared as follows: Deparaffinized tissue sections were rehydrated and incubated in 1X citrate buffer (pH6, Invitrogen Cat #00–5000) for 20 min using a DAKO PT Link. Then, with an Autostainer, the following steps and reagents were used for IHC analyses: (i) Incubation in 3% H_2_O_2_ for 15 min; (ii) incubation with 10% normal goat serum 10 min (Thermo Fisher #50062Z) 10 min; (iii) incubation with Avidin/Biotin block (Vector Labs Cat#SP-2001); (iv) incubation with individual primary antibodies diluted in 1% BSA for 30 min; (v) incubation with secondary goat anti-rabbit (Vector labs #BA-1000) for 15 min; (vi) incubation with ABC reagents (Vector Labs Cat #PK 6100) for 30 min; (vii) incubation with DAB substrates (Dako Cat #K3467) for 5 min; (viii) counterstained with DAKO Hematoxylin for 20s; and (ix) completing the final coversliped slides.

### Additional statistics analysis

Data were evaluated using the Student’s t-test or GraphPad Prism version 9. *P* ≤ 0.05 was considered statistically significant. Data are presented as means + SD as indicated. For all graphs: *P ˂0.05; **P˂0.01; ***P˂0.001; ****P˂0.0001.

## Results

### In vivo screening of FL7N1, FL7N2, and FL7N3 identified FL7N1 as the best FL118 analog

**Fig. 1:**
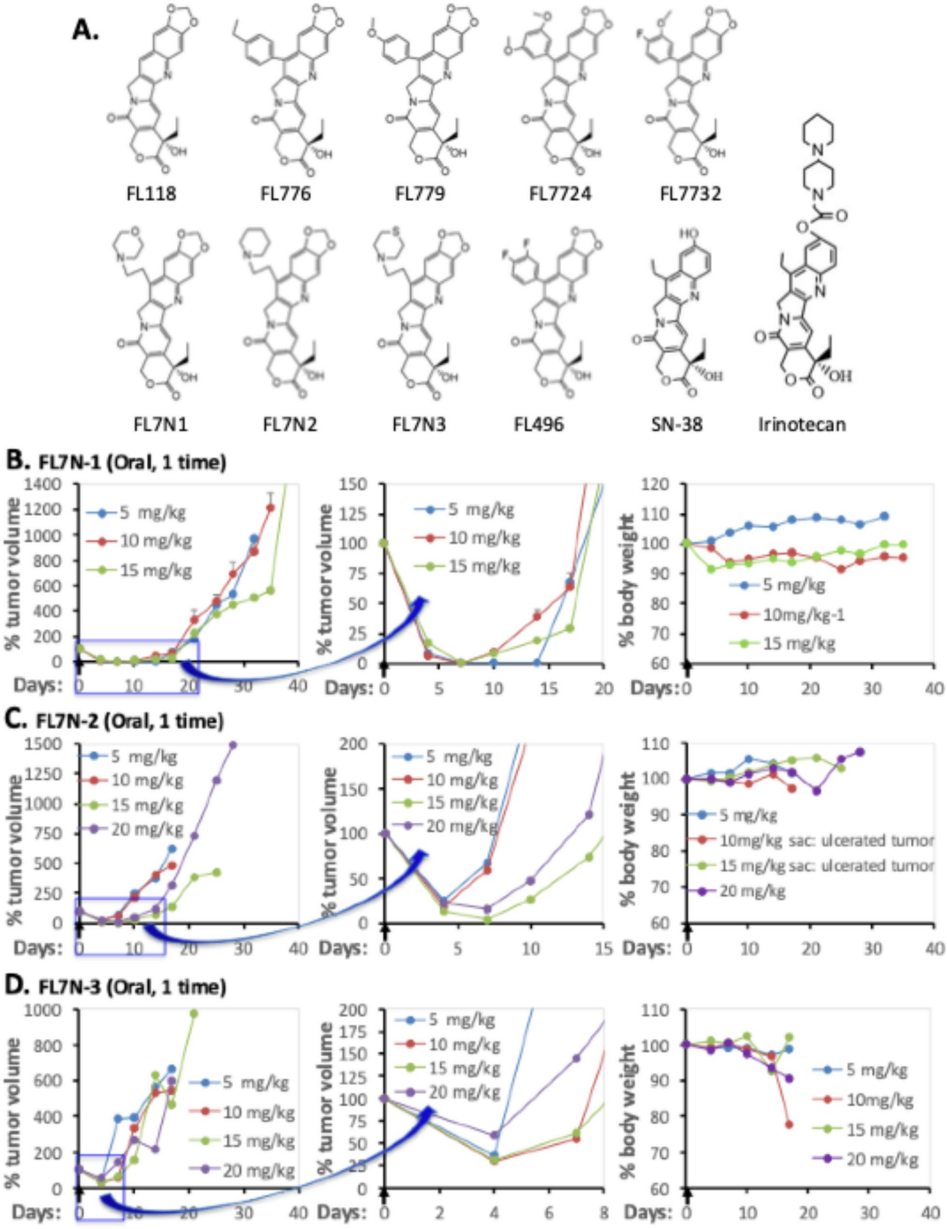
Determination of the efficacy and toxicity of FL7N1, FL7N2 and FL7N3 using human cervical cancer HT-3 cell-established tumor models. (**A**) Chemical structure of FL118, FL118-derived analogs (FL776, FL779, FL7724, FL7732, FL7N1, FL7N2, FL7N3, FL496) and two structure-relevant positive control drugs (SN38, irinotecan). Notably, SN38 is the active metabolite of irinotecan. In cell cultural in vitro studies, Irinotecan exhibits less than 10% of cell growth inhibitory activity in comparison with SN-38. (**B**, **C**, **D**) Use of the human HT-3 tumor animal model to determine antitumor efficacy and toxicity of FL7N1, FL7N2 and FL7N3. Human HT-3 tumor model setup: One hundred µL HT-3 cells (1 × 10^6^) with 50% Matrigel in a concentration of 1 × 10^7^ cells per mL were subcutenously injected in the area of flank area of SCID (severe combined immunodeficiency) mice. The established tumors passed at least one passage for tumor growth stabilization. HT-3 tumors were isolated from tumor maintenance mice and were used to implant experimental tumor mice using an implant needle (trocar) as detailed in the “Materials and Methods” section. HT-3 tumors were treated orally once (arrowed) with FL7N1, FL7N2 and FL7N3 in the doses shown. (**B**) Antitumor efficacy and body weight change curves from FL7N1-treated tumor mice. (**C**) Antitumor efficacy and body weight change curves form FL7N2-treated tumor mice. (**D**) Antitumor efficacy and body weight change curves from FL7N2-treated tumor mice. Each tumor and body weight change curve is within 10% and 1–2 mice were used for each drug level during the drug screening

In our previous research, we synthesized a series of benzene ring-based modifications of FL118 on Position 7 and identified several FL118 analogs that exhibit high antitumor efficacy with acceptable toxicity. This includes FL776, FL779 and FL7724 [20] as well as FL7732 (7 h) [21] as the top FL118 analogs for further studies (Fig. 1A). As shown in Scheme 1, we have chemically modified FL118 on Position 7 with a heterocyclic chemical group and obtained 3 heterocyclic FL118 analogs, FL7N1, FL7N2, and FL7N3 (Fig. 1A).

To identify the best one among the three novel FL118 analogs (FL7N1, FL7N2, FL7N3) containing heterocyclic rings for comparative studies in parallel with FL118 in MPM models system, we initially planned to use the most famous and widely used human cervical cancer HeLa cell line for our in vivo functional screening. However, the HeLa cell line is HPV-positive which may not be appropriate for our mesothelioma-focused studies that have nothing to do with HPV. We therefore used the HPV-negative human cervical cancer HT-3 cell line to initiate our in vivo efficacy screening of the three compounds.

To perform the test in a time/cost-effective manner and in an unbiased condition to compare the anti-HT-3 tumor activity and toxicity to animals, we used one oral administration of our formulated compound (FL7N1, FL7N2, or FL7N3) with multiple dosing levels as shown (Fig. 1BCD). Our human HT-3 tumor animal model studies indicated that after one-time treatment, only the FL7N1 could transiently eliminate HT-3 tumors in all three FL7N1 dosing levels (5, 10, 15 mg/kg, Fig. 1B), while neither FL7N2 or FL7N3 could eliminate HT-3 tumors at any treatment dosing levels (5, 10, 15, 20 mg/kg, Fig. 1CD). Importantly, FL7N1 showed no toxicity issues, whereas FL7N2 and FL7N3 each had some type of toxicity issue, including body weight loss (Fig. 1BCD, right panel). This in vivo study revealed that FL7N1 is the best candidate for further studies.

### FL496 was identified as the most effective drug to inhibit MPM cell growth/viability

Our previous studies indicated that FL7N1 has the potential to treat rare pediatric cancer retinoblastoma [24], and FL496 (Hx6) has the potential to treat the fumarate hydratase (FH) genetic defect papillary renal cell carcinoma (FHpRCC) [22], a specialized rare kidney cancer. To determine the relative potency and efficacy of FL7N1 with FL118 and FL496 (Scheme 1, Fig. 1A), we used the FHpRCC patient tumor-derived cell line UOK262. Our studies indicated that FL496 exhibited the most potency to inhibit cell growth and induce apoptosis (Supplemental Figure S3AB). Therefore, after confirming their IC50 in MPM cancer cell models, we used FL496 to delve deeper in the MPM cell/tumor model.

**Fig. 2:**
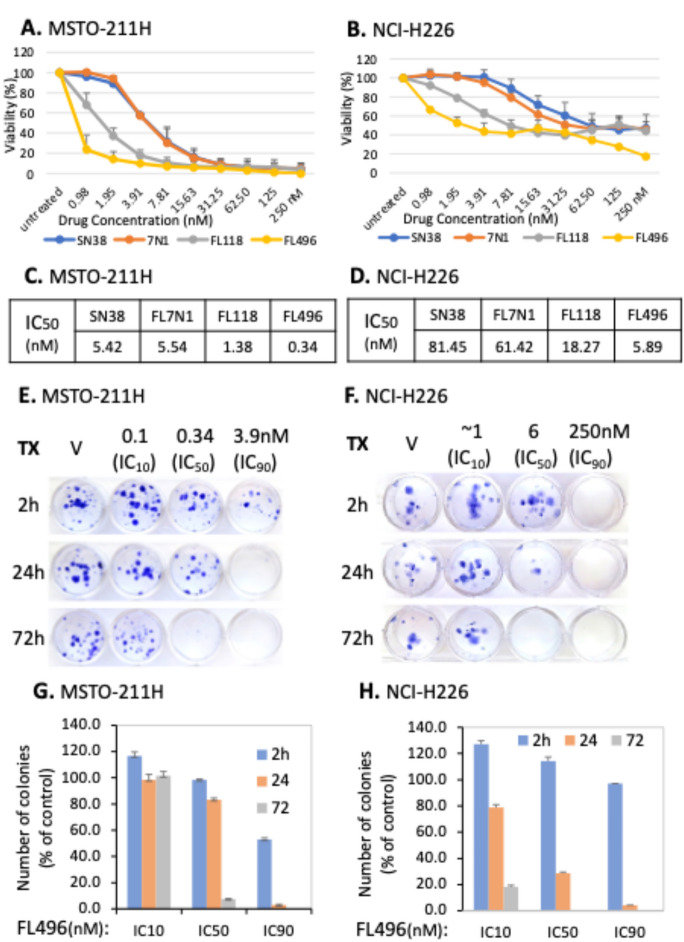
Malignant pleural mesothelioma (MPM) cells are more sensitive to FL496 than all other drugs tested. (**A**,** B**) Two MPM cell lines (MSTO-211H, NCI-H226) were seeded at 4 × 10^3^ per well in 96-well plates. After sitting overnight, cells in 96-well plates were treated with a concentration range (0 to 250 nM) of SN38, FL7N1, FL118 and FL496 for 72 h. Cell viability was determined using Resazurin viability (MTT) assay and was calculated as a percentage of untreated control. Each curve in A and B is the mean values + SD derived from triplicate. (**C**,** D**) MPM cell IC50 values for each of the drugs tested in A and B are presented. (**E**,** F**) Colony formation ability is strikingly reduced by FL496 treatment over time. Clonogenic assay was performed in 12-well plates with cell clones produced by MSTO-211H and NCI-H226 MPM cells. Cells were treated with IC_10_, IC_50_, and IC_90_ of FL496 for 2 h, 24 h, and 72 h. Then, colonies were allowed to form over 14 days without drugs before being stained with 0.5% Crystal violet. Colonies were then photographed and counted. (**G**,** H**) MPM cells colony formation statistical data derived from triplicate studies were presented in histogram, each bar is the mean + SD derived from triplicate

First, we performed a MPM cell growth inhibition study to compare FL496 with FL118 and FL7N-1 in parallel with the control drug SN-38. We found that among these small molecules, FL496 had the highest MPM cell inhibitory activity in both MSTO-211H (Fig. 2A) and NCI-H226 (Fig. 2B) cells making it the strongest candidate. The IC_50_ value presented in Fig. 2C and D further quantitatively confirmed this conclusion. Next, we determined the inhibitory effect of FL496 on the MPM cell colony formation and found that FL496 showed a concentration and treatment time-dependent inhibition of MSTO-211H and NCI-H226 colony formation. Examples of colony formation after FL496 treatment for each cell line are provided in Fig. 2E and F. The statistical results calculated from the triplicate experiments plotted in histograms are presented in Fig. 2G and H, respectively. Additionally, the FL496 treatment also changed the morphology of the MSTO-211H and NCI-H226 cells (Supplemental Figure S4A and S4C) and significantly attenuated the replicative cell growth capacity of these cells (Supplemental Figure S4B and S4D).

### FL496 activates p53, inhibits Rb and induces MPM cell senescence, G1 arrest and apoptosis

**Fig. 3:**
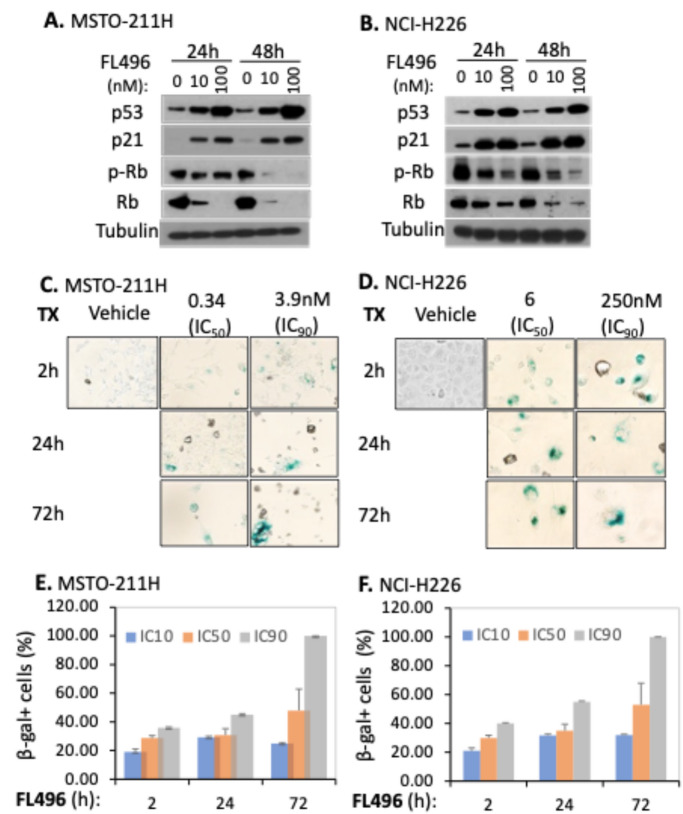
FL496 induces p53 activation and inhibits Rb phosphorylation and expression, which is associated with MPM cell senescence. (**A**, **B**) FL496 treatment induces p53 accumulation, Rb dephosphorylation and Rb depletion. Sub-confluent MSTO-211H (**A**) or NCI-H226 (**B**) cells were treated with FL496 as shown. Cells were then lysed for Western blot analysis using the corresponding antibodies. Tubulin expression is the internal control for total protein loading. (**C**, **D**) Exposure to FL496 for various time points (2 h, 24–72 h) is associated with a prevalence of cell senescence-associated β-galactosidase (SA-β-gal) positive cells compared to cells grown in the absence of FL496. Representative images of SA-β-gal staining in cells were shown for 4 days after FL496 removal. Green marks indicate SA-β-gal positive (senescent) cells. (**E**, **F**) Quantification analysis of the SA-β-gal positive cells of all the living cells are presented as the mean ± SD derived from 3 testing in duplicates

MSTO-211H and NCI-H226 possess wild-type p53 [35]. It is known that p53 and Rb proteins play essential roles in MPM cell fates. We determined the effect of FL496 on the expression of p53 and Rb proteins, as well as the Rb phosphorylation in both MSTO-211H and NCI-H226 MPM cells using Western blots. We found that FL496 treatment strikingly induces the accumulation of p53 protein in parallel with the induction of p21, a typical p53 downstream target (Fig. 3A and B), suggesting that FL496-induced p53 accumulation has activated p53 transcriptional function. It is well known that p53 induction/activation can induce cancer cell senescence and G1 arrest. Consistently, after FL496 treatment we obtained large and flat MPM cells mixed with condensed dead cells over time, and the large flat cells exhibited β-gal positivity in the senescence-associated β-gal staining assay (Fig. 3C and D). The statistical β-gal positive cells calculated from the triplicate experiments are presented in the histogram (Fig. 3E and F).

Next, we studied the MPM cell cycle distribution after FL496 treatment. Consistent with the notion that activation of p53 could arrest cells in the G1 phase, propidium iodide (PI)-staining and flow cytometry analysis of FL496-treated MSTO-211H cells by gating the living and senescent cells indicated that after FL496 treatment, the living and senescent cells are arrested in G0/G1 phases (Supplemntal Figure S5A). Similar results were also obtained from NCI-H226 MPM cells (Supplemental Figure S5B). In parallel, we determined the MPM cell DNA fragmentation (apoptosis) using a DNA fragmentation cell death ELISA assay kit. This study revealed that after FL496 treatment, significant DNA fragmentation/apoptosis was enhanced by FL496 (Supplemental Figure S5C-F).

### MPM cells with p53 knockout (KO) are less sensitive to FL496 than the parental cells

**Fig. 4:**
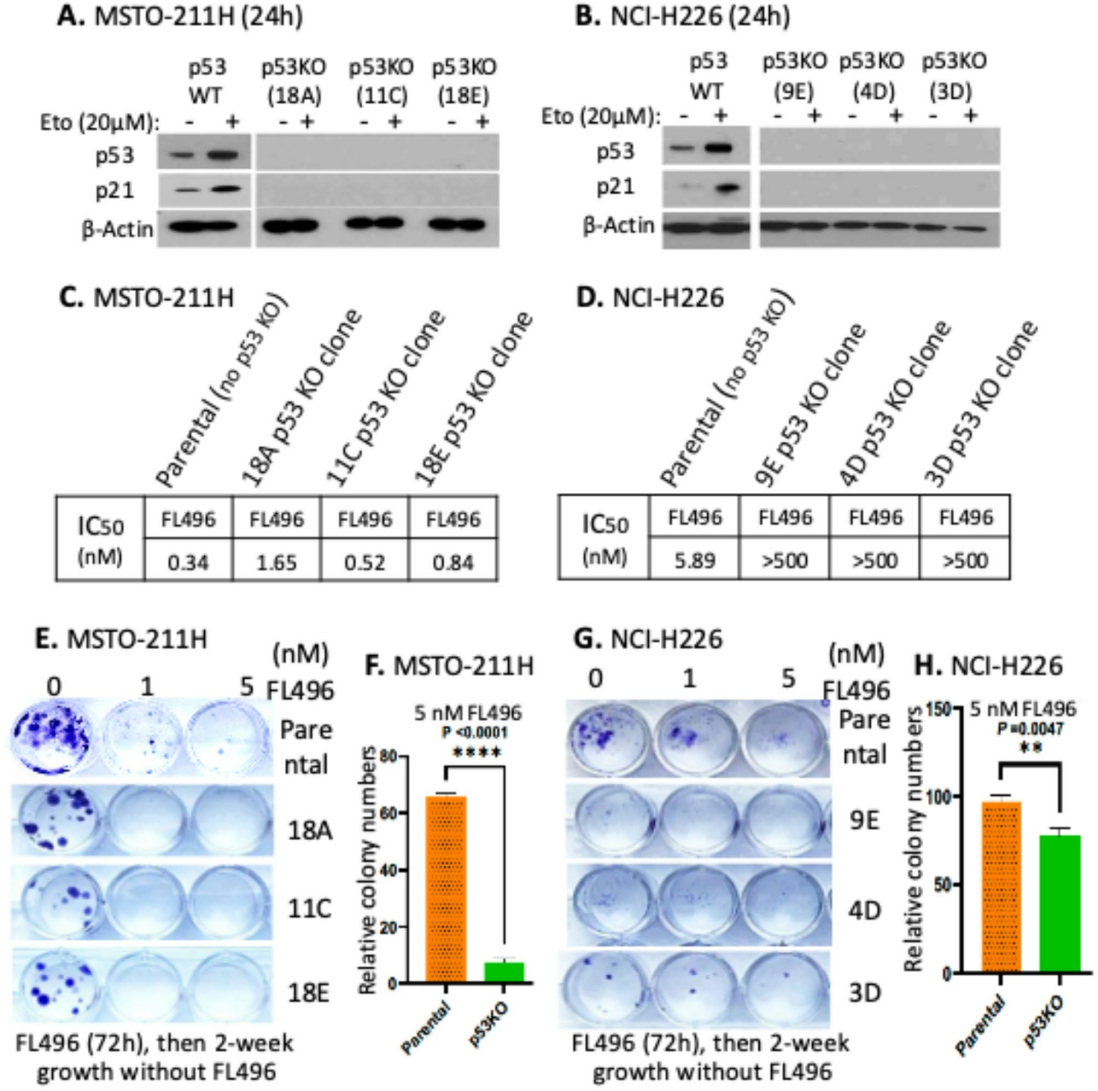
Knockout (KO) of TP53 (p53 gene) in MPM cells increases FL496 IC_50_ in comparison with no p53 KO parental MPM cells. (**A**, **B**) Confrmation of TP53 KO in 3 independent p53 KO cell clones by the induction of p53 and p21 expression in parental and p53 KO MPM cells using Western blot analysis. (**C**, **D**) IC_50_ comparison between (no p53 KO) parental MPM cells and p53 KO MPM cells. Parental MPM cells (MSTO-211H and NCI-H226) versus p53 KO MSTO-211H cell clones (18 A, 11 C, 18E) and p53 KO NCI-H226 cell clones (9E, 4D, 3D) at 4 × 10^3^ per well were seeded in 96-well plates. After sitting overnight, cells in 96-well plates were treated with a series of FL496 concentations (0, 0.08, 0.23, 0.69, 2.1, 6.17, 18.52,55.56, 166.67 and 500 nM) for 72 h. Cell viability was determined using MTT assay. Then, the FL496 IC_50_ nM value for each cell clone was calculated based on the MTT MPM cell viability data (FL496 IC_50_ value of the parental MPM cells from the data shown in Fig. 2C and D). (**E**) Representative images from the colony formation assay studies are shown. Large colonies were formed in two weeks, suggesting cell growth is not very slow. (**G**) Representative images from the colony formation assay studies are shown. More small colonies were formed in two weeks, suggesting cell growth is very slow. (**F**, **H**) The histogram shows relative cell colony numbers quantified at the end of colony formation assay studies derived from multiple plate wells after colony average per well

Since p53 plays an important role in mesothelioma, we determined the role of p53 in FL496-mediated MPM cell growth inhibition. To do this, we used the vector-free Crispr-Cas9 technology to knock out TP53 in MSTO-211H and NCI-H226 MPM cells. Through single cell clone selection, genetic sequencing (Supplemental Figures S1, S2) and Western blot analysis, we identified 3 MSTO-211H cell clones with TP53 KO (11 C, 18 A, 18 E) and 3 NCI-H226 cell clones with TP53 KO (3D, 4D, 9E). We further confirmed the TP53 KO with and without 20 µM DNA-damage drug etoposide (Eto) treatment of parental and TP53 KO MPM cells, followed by Western blot analysis of p53 and p21 expression. As expected, Eto-treated cells showed the induction of the expression of p53 and p21 (p53 downsream target) in parental MPM cells, while this did not happen in TP53 KO cells (Fig. 4AB). Next, to determine FL496’s effects on MPM MSTO-211H cell growth/viability inhibition in the context of MPM cells with p53 versus without p53 (i.e., 53 KO), we utilized the identified p53 KO MSTO-211H MPM cells in parallel with no p53 KO parental MPM MSTO-211H cells and perfomed MTT cell viability assays after treatment using a series of FL496 concentrations (0–500 nM) for 72 h. We found the p53 KO MPM MSTO-211H cells exhibited less sensitive than the parental MPM MSTO-211H cells with p53 as indicated by their corresponding IC_50_ value (Fig. 4C). Interestingly, although data were consistent, the NCI-H226 cells with p53 KO strikingly increased their IC50 valuse; that is, FL496 at 500 nM didn’t achieve IC_50_ values (Fig. 4D). Next, we performed cell colony formation assay for these p53 KO MPM cell clones to evaluate the colony-forming ability of p53 KO MPM cells versus no p53 KO parental MPM cells with and without FL496 treatment (Fig. 4EG). We found that treatment as low as 1 nM of FL496 for 24 h can diminish the ability of the p53 KO MSTO-211H cell colony formation in 2-week cell culture for all three p53 KO MSTO-211H cell clone lines (18 A, 11 C, 18E) compared to their parental MSTO-211H (Fig. 4E). Similar colony formation results were obtained from p53 KO NCI-H226 cells (9E, 4D, 3D) versus (no p53 KO) parental NCI-H226 cells but with much less sensitivity (Fig. 4G). The quantified histogram cell colony number formation at the 5 nM FL496 treatment condtion averaged from multiple plate wells is shown in Fig. 4F and H. Together, our studies indicated that p53 KO MSTO-211H and NCI-H226 cells are less sensitive to FL496 treatment than their corresponding no p53 KO parental MPM cells, although p53 KO NCI-H226 cells are much more resistance to FL496 treatment in comparison with p53 KO MSTO-211H cells. These studies suggest that p53 plays at least a partial role in FL496 efficacy to inhibit MPM cell growth.

### FL496-induced γH2AX accumulation (DNA damage marker) is associated with p53 and Ser15-phosphorylated p53 accumulation

**Fig. 5:**
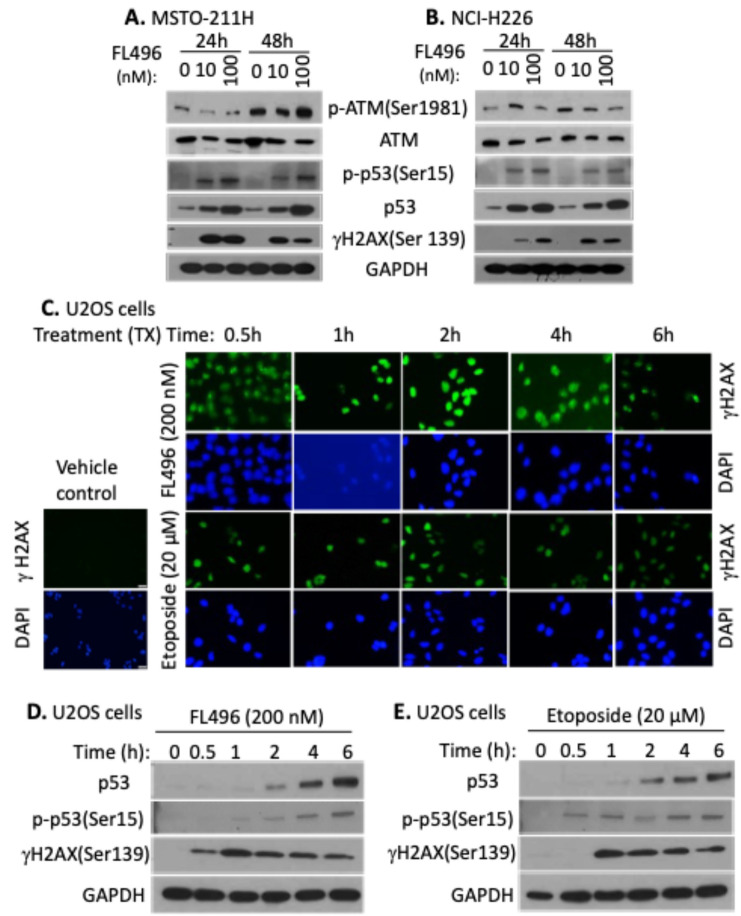
FL496 rapidly induces DNA damage in MPM cells. (**A**,** B**) γH2AX expression is increased in MPM cells after FL496 treatment. Western blots showed the expression modulation profile of p-ATM(S1981), ATM, p-53(S15), p53 and γH2Ax(S139) in response to FL496 treatment (10 nM, 100 nM) for 24 h and 48 h. GAPDH is the internal control for total protein loading. (**C**) Alternative demonstration of DNA damage marker γH2AX accumulation in U2OS cells using immunofluorescence microscopy techniques. Representative fluorescence microscopic images are shown as increased nuclear localization of γH2AX (green) in cells in exposure to FL496 or etoposide. Nuclei are counterstained with DAPI (blue). (**D**, **E**) Time course of the induction of p53, p-p53 and γH2AX expression by FL496 (**D**) or etoposide (**E**) treatment over time in U2OS cells. Sub-confluent U2OS cells were treated with FL496 (**D**) or etoposide (**E**) for a series of time points (0 h to 6 h) as shown. Cells were then lysed for Western blot analysis with their corresponding antibodies. GAPDH expression is the internal control for total protein loading

Next, we assessed the expression of the DNA damage marker γH2AX along with DNA repair-relevant proteins, ATM, p-ATM(S1981), p53, and p-p53(S15). Our Western blot studies indicated that FL496 treatment strikingly enhances γH2AX(S139), p53 and p-p53(S15) levels in MSTO-211H (Fig. 5A) and NCI-H226 (Fig. 5B) cells without significantly affecting the expression of ATM and p-ATM(S1981) levels (Fig. 5AB). To further confirm the results obtained with MPM cells, we alternatively determined the time course of γH2AX induction by FL496 and Etoposide (positive control) in the osteosarcoma U2OS cells. Consistent with the data shown in Fig. 5AB, our immunofluorescence studies indicated that FL496 rapidly induced γH2AX (S139) foci in the nuclei in as short as 30 min (Fig. 5C). Furthermore, our Western blot time-course studies indicated that FL496-induced γH2AX expression occurs much earlier (0.5 h) than FL496-induced p53 and p-p53(S15) expression (~ 2 h) (Fig. 5D). This indicated that FL496-mediated induction of γH2AX expression through signaling transduction pathways is p53-independent (see more data below). This observation supports the finding that KO of p53 makes FL496 more effective to kill cancer cells. Interestingly, in contrast to FL496, the topoisomerase II inhibitor, etoposide induces p-p53(S15) in as short as 30 min; then induces γH2AX and p53 (Fig. 5E), suggesting FL496 uses a mechanism of action (MOA) that is different from etoposide (See Descussion section).

**Fig. 6:**
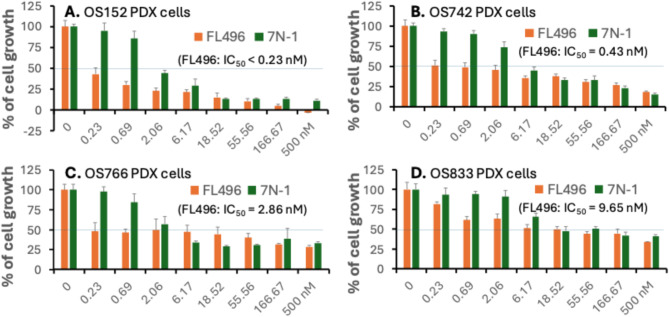
Pediatric osteosarcoma PDX tumor-established cell lines exhibited high sensitivity to FL496 treatment. Cells were seeded at 4 × 10^3^ per well in 96-well plates. After overnight incubation, cells in 96-well plates were treated with a range of concentrations (0 to 500 nM) of FL496 and FL7N1 (comparason control) for 72 h. Cell growth/viability was determined using Resazurin viability (MTT) assay and was calculated as a percentage of untreated control. Each curve is the mean values + SD derived from triplicate. (**A**) The OS152 cell line is a metastasis type with Myc amplification, which was derived from a 11-year osteosarcoma female patient’s scapula (tissue) site of femur (primary). (**B**) The OS742 cell line is a diagnostic type with Myc and CDK4 amplification, which was derived from a 10-year osteosarcoma female patient’s tibia (primary and tissue) site. (**C**) The OS766 cell line is a metastisis type with CDK4 amplificaiton, which was derived from a 30-year osteosarcoma female patient’s lung (tissue) site of femur (primary). (**D**) The OS833 cell line is a resection type with Myc amplification, which was derived from a 14-year osteosarcoma male patient’s fibula (primary and tissue) site

To alleviate the limitation of using two mesothelioma cell models and to further strengthen our overall studies in the area of rare cancer, while mainly focusing on both in vitro and in vivo mesothelioma studies, on the basis of the osteosarcoma U2OS cell data shown in Fig. 5CDE, we used additional four pediatric osteosarcoma patient-derived xenograft (PDX) tumor-established cell models to functionally determine the FL496 efficacy on such model cell growth/viability inhibition. Our data indicated that FL496 exhibited high efficacy to inhibit such cell growth/viability (Fig. 6, FL7N-1 as a comparison control). This result has provided an intriguing possibility that FL496 may also be a superior candidate to develop into a new option for pediatric osteosarcoma patient treatment.

### FL496 inhibits the expression of Mcl-1, Bcl-2, and Bcl-XL, which are associated with activation of apoptosis hallmarks (caspase-3, PUMA, PARP cleavage)

**Fig. 7:**
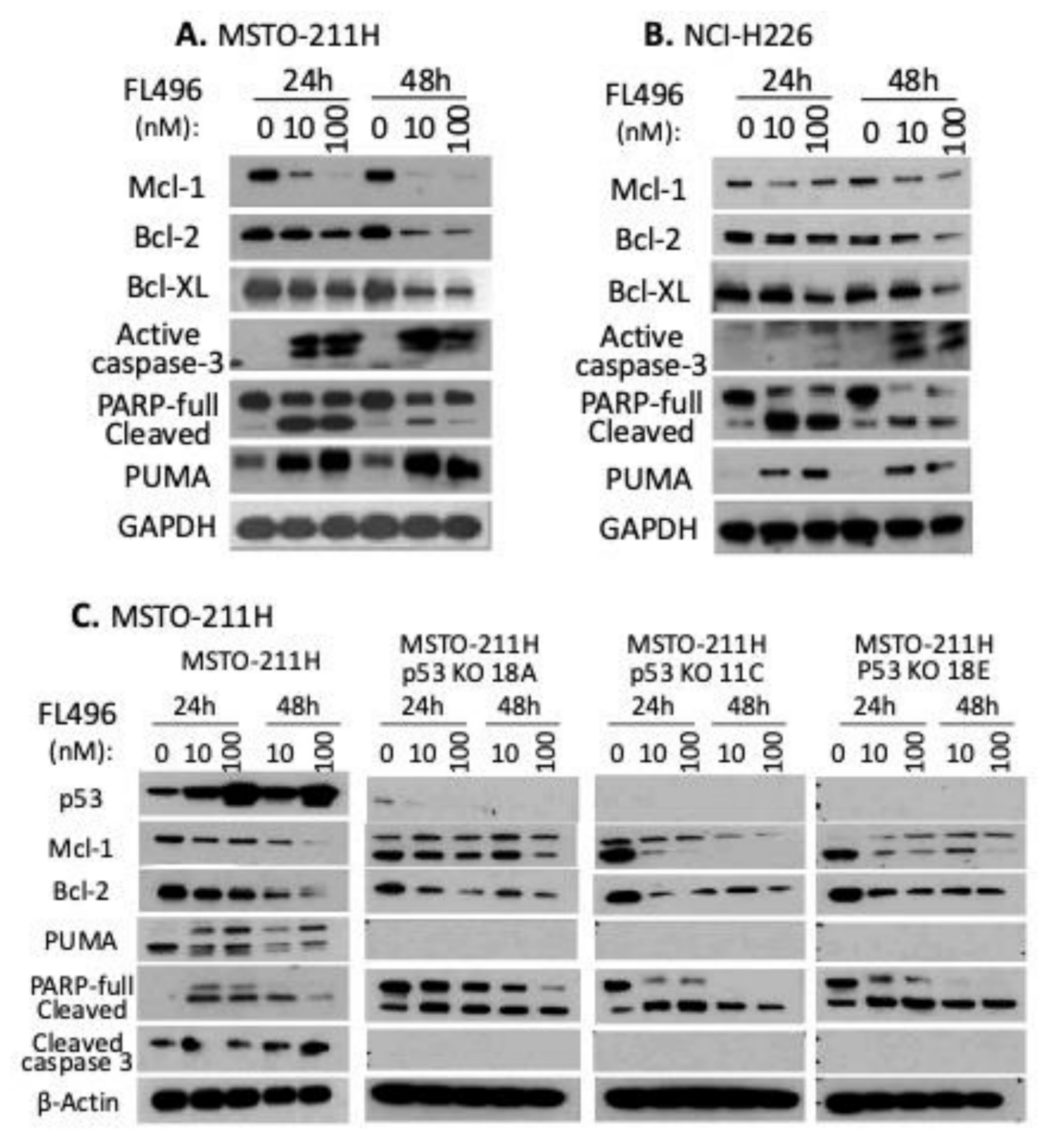
Inhibition of Mcl-1, Bcl-2 and Bcl-XL by FL496 is associated with the induction of apoptotic markers (caspase-3 cleavage/activation, PARP cleavage, PUMA induction). (**A**,** B**) FL496 treatment inhibits anti-apoptotic protiens but induces pro-apoptotic proteins in MSTO-211H (A) and NCI-H226 (B) MPM cells. Sub-confluent MSTO-211H (A) or NCI-H226 (B) cells were treated with FL496 as shown. Then cells were lysed for Western blot analysis using the corresponding antibodies. GAPDH expression is the internal control for total protein loading. (**C**) FL496 treatment-induced modulation of anti-apoptotic and pro-apoptotic proteins in the parental MSTO-211H cells versus in the p53-KO NCI-H226 cell clones (11 C, 18 A, 18E). Sub-confluent MPM cells were treated with FL496 as shown. Cells were then lysed for Western blot analysis using the corresponding antibodies. β-Actin expression is the internal control for total protein loading

Anti-apoptotic/pro-survival BH3-family members, Bcl-2, Bcl-xl, and Mcl-1 are overexpressed in MPM samples and cells [36–39], which suggests that MPM cell survival depends on the expression of these proteins to evade apoptosis and promote cell survival [40]. To further study the mechanism by which FL496 induces MPM cell growth inhibition and apoptosis induction, we determined the expression of Bcl-2 family antiapoptotic proteins Mcl-1, Bcl-2, Bcl-XL with and without FL496 treatment. We found that all of these pro-survival proteins were suppressed in a dose and time-dependent manner in MSTO-211H (Fig. 7A). Similar results were obtained using NCI-H226 cells (Fig. 7B). Concurrently, the inhibition of Bcl-2 family antiapoptotic proteins was associated with an increase in activated/cleaved Caspase-3, cleaved PARP, and upregulation of the p53-regulated pro-apoptotic protein PUMA (Fig. 7AB), indicating MPM cells had gone into apoptosis.

To further investigate the expression profiles of Mcl-1, Bcl-2, PUMA, cleaved PARP and cleaved caspase-3 in parental MSTO-211H cells compared to p53-KO MSTO-211H cells, both cell types were treated with FL496 (10 and 100 nM) or vehicle control for 24 h and 48 h, followed by Western blot analyses using their corresponding antibodies. We found that p53 KO cells still showed Mcl-1 and Bcl-2 downregulation after FL496 treatment. However, PUMA and cleaved caspase-3 (both are p53 downstream gene targets) disappeared after p53 KO in pMSTO-211H cells (Fig. 7C). These results indicate that inhibiton of Mcl-1, Bcl-1 and induction of PARP cleavage is p53-independent, while induction of PUMA and caspase-3 cleavage are p53-dependent.

### High survivin expression is associated with shorter MPM patient survival, and DDX5 inhibition by FL496 is associated with survivin inhibition in MPM cells

**Fig. 8:**
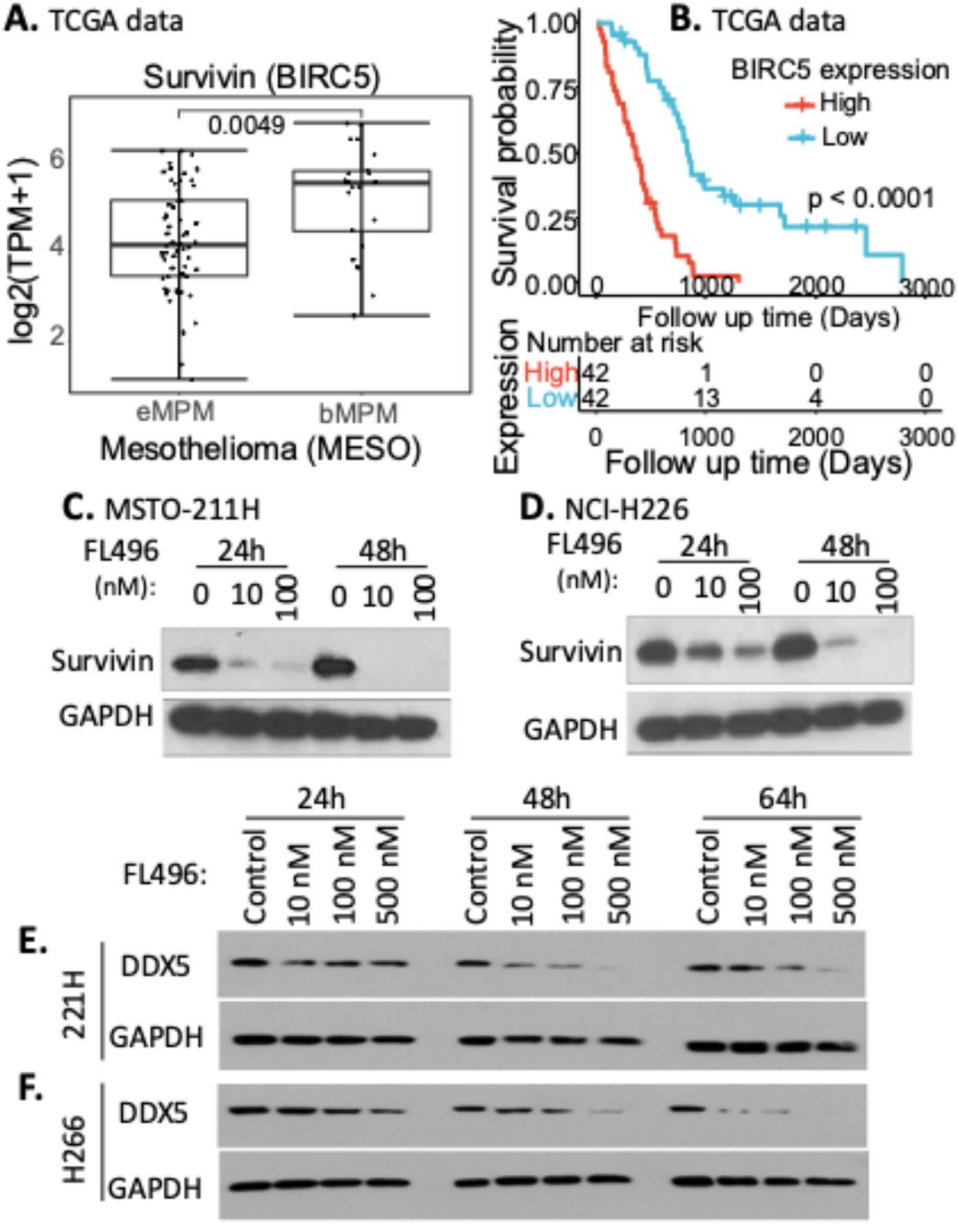
High expression of survivin is assoicated with poor MPM patient survival. (**A**) Survivin (BIRC5) expression comparison between epithelioid MPM (eMPM) and biphasic MPM (bMPM) from the TCGA mesothelioma (MESO) dataset in the boxplot format. BIRC5/survivin expression was presented in the log2 (TPM + 1) scale format. (**B**) Low survivin (BIRC5) expression on MPM patients’ tumor is associated with better patient survival. Kaplan-Meier survival analyses of overall survival from TCGA-MESO cohorts were shown. (**C**,** D**) Treatment of MPM cells with FL496 strongly inhibits the expression of survivin. Sub-confluent MSTO-211H (C) or NCI-H226 (D) cells were treated with FL496 as shown. Cells were then lysed for Western blot analysis using survivin antibodies. GAPDH expression is the internal control for total protein loading. (**E**,** F**) FL496 treatment of MPM cells reduces the expression of DDX5. Sub-confluent MSTO-211H (E) or NCI-H226 (F) MPM cells were treated with FL496 for 24 h, 48 h and 64 h as shown. Cells were then lysed and used for Western blot analysis with DDX5 antibodies. GAPDH expression is the internal control for total protein loading

It was shown that survivin is highly expressed in MPM and that the inhibition of survivin expression by survivin siRNA causes the decline in cell growth and the induction of apoptosis in MPM cells [41]. MPM can be classified into three major histological subtypes, epithelioid MPM (eMPM), sarcomatoid MPM (sMPM), and the mix of the two subtypes called biphasic MPM (bMPM), which contains both epithelial and spindle-shaped cells [42]. Consistent with the known fact that bMPM is more aggressive than the eMPM, our bioinformatic analysis of survivin expression in bMPM is significantly higher than those in eMPM (Fig. 8A), although survivin expression across various stages of MPM (stage i-iv) or among female and male patients did not show significant differences (Supplemental Figure S6AB). Importantly, high survivin expression is strongly associated with shorter MPM patient survival (Fig. 8B). To determine whether FL496 treatment of MPM cells could result in survivin downregulation, MSTO-211H and NCI-H266 MPM cells were treated with FL496 and analyzed using Western blots. Our studies indicated that FL496 strongly inhibits survivin expression in both MSTO-211H MPM cells (Fig. 8C) and NCI-H226 MPM cells (Fig. 8D). Thus, high survivin expression may play a role in both MPM patient shorter survival and a direct or indirect target of FL496 in FL496-mediated MPM cell growth inhibiton and apoptosis induction.

Our recent studies indicated that FL118 directly binds to and functionally degrades DDX5 protein, and survivin is one of the DDX5 downstream targets [14]. Therefore, after MSTO-211H and NCI-H266 MPM cells were treated with FL496, the cell lysates were analyzed using Western blots to determine whether FL496 could reduce DDX5 expression. Our studies revealed that, like FL118, FL496 can reduce DDX5 expression in both MSTO-211H MPM cells (Fig. 8E) and NCI-H226 MPM cells (Fig. 8F). Thus, the high efficacy of FL496 may also use the FL118 direct target DDX5 pathway as one of its effector target signaling pathways to execute its function.

### FL496 alone demonstrates significantly greater anti-MPM tumor compared to the pemetrexed-cisplatin combination

**Fig. 9:**
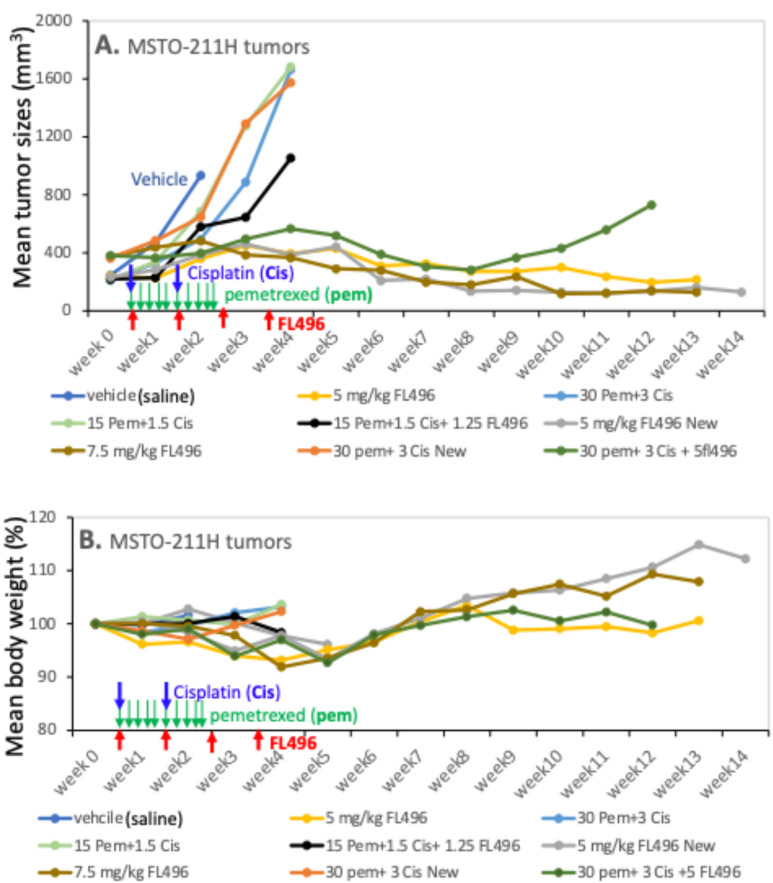
Anti-MPM tumor efficacy and toxicity (body weight changes) of FL496 versus the pemetrexed plus cisplatin as well as the three drug combination: MPM MSTO-211H xenograft tumor model setup in SCID mice and drug treatment were carried out as described in the “Materials and Methods” section. As shown in A and B, the schedule of FL496 was weekly x 4 (red arrows); the schedule of pemetrexed was five times per week for two weeks (green arrows); and cisplatin weekly x 2 (blue arrows), which matches the previous studies in the literature. FL496, pemetrexed and cisplatin were intraperitoneally administered. (**A**) Tumor growth curve changes over time after various treatments marked with different color arrows as shown. Each tumor growth curve is the mean tumor size derived from 5 tumors on 5 SCID mice (tumor size variation was within 15%). (**B**) SCID mouse body weight curve changes over time after various treatments arrowed with different colors as shown. Each body weight change curve is the mean body weight derived from 5 SCID mice body weights (body weight change variation was within 10%). For clarity, we did not add SD variations in Fig. 10A or 10B

A phase III study with MPM patients indicated that pemetrexed-cisplatin combination shows superior survival time in comparison with cisplatin alone (12.1 months versus 9.3 months), time to progression (5.7 months versus 3.9 months), and response rates (41.3% versus 16.7%) [43–45]. After reviewing the use of pemetrexed and cisplatin in the dose level and schedule for preclinical animal model studies [31–34], we designed a comprehensive in vivo study for a comparison of FL496 alone with pemetrexed-cisplatin combination as described in the “Materials and Methods” and indicated with blue, green and red arrows in Fig. 9. Our studies found that FL496 alone exhibited significantly greater anti-MPM tumor efficacy than the pemetrexed-cisplatin combination (Fig. 9A). Intriguingly, while pemetrexed-cisplatin combination plus a low dose level of FL496 could significantly impove the anti-MPM tumor efficacy from pemetrexed-cisplatin combination, pemetrexed-cisplatin combination plus FL496 did not show a better anti-MPM tumor efficacy than FL496 alone (Fig. 9A). Furthermore, while all treatment groups showed no significant toxicity including animal body weight changes (within 10%), pemetrexed-cisplatin combination plus FL496 at the comparable dosing levels showed more animal body weight loss than those from FL496 alone (Fig. 9B). These intriguing in vivo results imply that FL118 may have high potential to be developed into monotherapy for MPM patients.

### FL496 induces MPM tumor cell apoptosis, DNA damage and cell proliferation inhibibiton in vivo

**Fig. 10:**
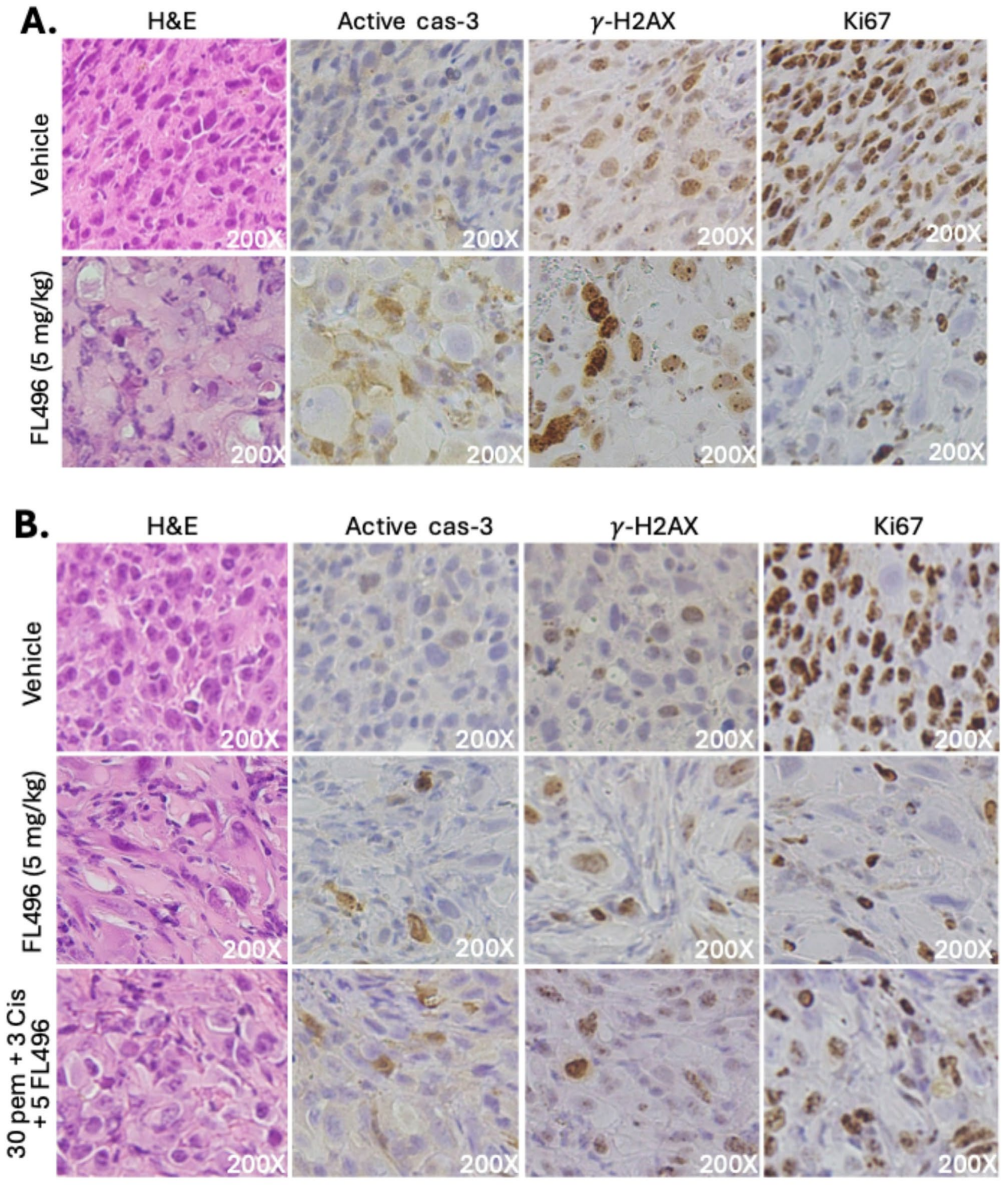
FL496 increased the expression of active caspase-3 (cas-3) and γH2AX, and decreased Ki67 in human MPM tumors collected from FL496-treated mice. Representative images were shown for the 4-week timepoint (**A**) and 8-week time point (**B**)

To evaluate whether FL496 induces apoptosis and DNA damage and inhibits cell proliferation in MPM tumors, we treated MPM tumor-bearing mice with FL496 alone or in combination with pemetrexed (30 mg/kg) + cisplatin (3 mg/kg) using the same drug treatment schedule as the data shown in Fig. 9. Tumor tissues were collected at weeks 4 and week 8 for immunohistochemistry (IHC) analysis of protein markers associated with apoptosis, DNA damage and cell proliferation. Consistent with the in vitro findings shown in Fig. 7AB, FL496-treated tumors exhibited increased expression of active caspase-3 and γH2AX, and decreased expression of Ki67 at both time points (Fig. 10A and B).

Furthermore, MPM tumors collected from FL496-treated mice also showed decreased expression of antiapoptotic proteins, survivin and Bcl-XL at both time points (Supplemental Figure S7A and Supplemental Figure S7B). These IHC findings, presented in Fig. 10 and Supplemental Figure S7, are further discussed in the “Discussion” section.

## Discussion

Malignant Pleural Mesothelioma (MPM) is an aggressive and rare cancer that develops in the pleural cavity’s mesothelial cell lining which has been found to be causally associated with asbestos exposure in many patients. MPM responds poorly to chemotherapy and is a rapidly progressing malignancy with a short median survival time (6 to 9 months). Most patients are diagnosed at later stages when surgery is no longer an option. Furthermore, the most prevalent pemetrexed-cisplatin combination chemotherapy approved by the FDA about 20 years ago [43–45] showed modest benefits in overall survival but is still used as a major standard care for MPM patients [1], despite some promising outcomes from clinical trials using different combination regimens [46, 47]. Therefore, developing novel anti-MPM therapies with high efficacy and low toxicity is essential to meet the needs of MPM patients.

In this study, we first used cervical cancer HT-3 cell-established tumor models and identified the best candidate (i.e., FL7N1) among three heterocyclic chemical group-derived FL118 position 7 analogs (Fig. 1). As we previously mentioned, we used HT-3 instead of the more commonly used HeLa in the initial screening of the three heterocyclic ring compounds (FL7N1, FL7N2, FL7N3) because HeLa cells are HPV-positive and our mesothelioma-focused studies have nothing to do with HPV. After identifying FL7N1 as the best candidate for further studies, we used MPM cancer cell models and compared the top candidates (FL118, FL7N1, FL496) in parallel with positive control drug SN38 (the active metabolite of irinotecan) for their potency (IC_50_) and identified FL496 as the most potent candidate that exhibits much higher anti-MPM cell growth activity than FL7N1 and the extensively investigated FL118 (Fig. 2). Our studies indicated that treatment of MPM cells with FL496 rapidly induced the accumulation of p53 and p21 as well as decreased Rb and p-Rb, which are associated with MPM cell senescence and cell G_1_/G_0_ arrest (Fig. 3, Supplemental Figure S5AB). Previous studies indicated that TP53 mutation plays a role in the aggressiveness of MPM and is associated with poor prognosis [7, 8]. Consistently, our studies indicated that knockout (KO) of TP53/p53 gene increases the FL496 IC50 value (Fig. 4CD). This result suggests that p53 at least plays a partial role in FL496 inhbition of MPM cell growth in addition to the role of p53 in the FL496-mediated induction of G_1_/G_0_ arrest and DNA fragmentation (Supplemental Figure S5) as well as MPM cell senescence (Fig. 3CDEF).

Interestingly, while our previous studies found that FL118 could activate the p53 signaling pathway and induce colorectal cancer (CRC) cell senescence [48], FL118 exhibited higher efficacy to induce apoptosis and CRC cell death in the CRC cells with mutant p53 or without p53 (p53 null) [48]. In contrast, our studies in the current report found that KO of p53 in MPM cells increases FL496 IC_50_ values (i.e., decreases FL496-mediated inhibiton of MPM cell growth). This observation along with other findings presented in this report suggest that the mechanism of action (MOA) of FL496 is likely overlaps with but extends FL118 MOA.

While a deeper and extensive exploration of the MOA for FL496 will be required in our follow-up studies, we new use currently available data and the known MOA of FL118 to discuss the potential MOA for FL496. Our recent studies revealed that FL118 binds to the multifunctional oncogenic protein DDX5 (also called p68), and then induces the dephosphorylation and degradation of DDX5 in both CRC cells and pancreatic ductal adenocarcinoma (PDAC) cells [14]. We demonstrated that DDX5 promotes many downstream targets including survivin, Mcl-1, XIAP and cIAP2 [14], which were shown to be inhibited by FL118 [9]. Existing literature on DDX5 studies indicated that DDX5 controls multiple cancer cell functional processes including the function to repair damaged DNA in cancer cells [49]. Consistent with the observations from FL118 and DDX5, we demonstrated in this report that treatment of MPM cells or the rare cancer osteosarcoma U2OS cells with FL496 treatment rapidly induced DNA damage marker γH2AX prior to p53 activation (Fig. 5). In this regard, accumulation of p53 induced by FL496 treatment may potentially facilitate the repair of damaged MPM cell DNA by DDX5 which may be a cancer resistance mechanism. Since the functional relationship of DDX5 and p53 in MPM cells is currently unclear, additional studies to elucidate the mechanism by which p53 KO could decrease FL496 ability to inhibite MPM cell growth (i.e., increase FL496 IC_50_ values, Fig. 4CD) will be required.

To alleviate the limination of using two MPM cell models and to further strengthen our overall studies in the area of rare cancer, we determined whether FL496 could induce similar effects on p53, phosphor-p53 and γH2AX in osteosarcoma (another rare cancer) cells as was oberserved in MPM cells. We obtained consistent results in the osteosarcoma cell models (Fig. 5CDE) as those obtained in the mesothelioma cell models (Fig. 5AB). Furthermore, we have tested the FL496 efficacy in the inhibition of 4 additional osteosarcoma PDX tumor-established cell lines and found that FL496 exhibited high efficacy to inhibit osteosarcoma PDX tumor-established cell growth (Fig. 6). We previously reported the high efficacy of FL496 against the rare cancer fumarate hydratase (FH)-deficient papillary renal cell carcinoma (FHpRCC) [49]. These consistent findings suggest that FL496 may also hold strong therapeutic potential against other rare cancer types, which are supported by the osteosarcoma case studied in this report. However, this requires further efforts and additional investigation before definitive conclusions can be safely drawn.

Additionally, FL496 treatment of MPM cells inhibited multiple antiapoptotic proteins (e.g., Mcl-1, Bcl-2, survivin) and induced multiple apoptotic indicators/hallmarks (e.g., active/cleaved caspase-3, cleaved PARP and PUMA) (Fig. 7AB). Consistent with the fact that PUMA expression is p53-dependent, p53-KO MPM cells showed no PUMA exprsssion (Fig. 7C). However, the intriguing point that requires further investigation is that while p53 KO in MPM cells decrease FL496’s ability to inhibit MPM cell growth (i.e., increase FL496 IC_50_ values) and colony formation (Fig. 4C-H), p53 KO MPM cells showed no cleaved caspase-3 (Fig. 7C). This observation suggests that FL496 increasing p53-KO MPM cell growth inhibtion and cell death is at least partially independent of caspase-3 activation.

Recently, we synthesized 48 FL118 derivatives and through in vitro and in vivo studies, we identified 3 FL118 analogues (FL776, FL779, FL7724, Fig. 1A) that exhibited better anti-CRC patient-derived xenograft (PDX) and anti-PDAC PDX tumors. However, they showed the MOA similar to FL118 through control of DDX5 downstream targets and functions [20]. In this regard, our studies with MPM cell models indicated that FL496 could reduce DDX5 expression (Fig. 8EF). It is possible that the downregulation of DDX5 by FL496 plays a role in blocking damaged DNA repaired by DDX5 in MPM cells. This notion is consistent with our recent review on DDX5 indicating that DDX5 has multifaceted functions including repairing damaged DNA in cancer cells and promoting multiple oncogenic gene/protein expressions including survivin [49]. Accordingly, consisitent with the previous finding that survivin is highly expressed in mesothelioma cells [41], our bioinformatics analysis of TCGA data indicated that survivin expression is significantly higher in more aggressive bMPM than in the less aggressive eMPM (Fig. 8A). Furthermore, the high expression of survivin in MPM patients’ tumors is significantly associated with worse overall survivival versus mesothelioma patients with low survivin expression in their mesothelioma tumor (Fig. 8B). We noted that Hmeljak et al.’s report did not find the association of survivin expression with mesothelioma patients’ overall survival in their used patient cohort data [50]. However, consistant with our finding, the study reported by Zhang et al. found that a lower level of survivin expression is strongly associated with improved overall survival in grade 4 mesothelioma patients [51]. Thus, our analysis using TCGA datasets and a simple classification method for high versus low surviving expression (Fig. 8B) confirms the association between survivin levels and overall survival in mesothelioma patients. Furthermore, FL496 effectively inhibits the epxression of survivin in both MSTO-211H and NCI-H226 MPM cells (Fig. 8CD). Consistant with these observations, our in vivo MPM tumor animal model studies indicated that a schedule of weekly x 4 treatment of MPM tumors with FL496 alone could result in a long-term inhibition of MPM tumor growth and exhibited much better anti-MPM efficacy than those from the pemetrexed-cisplatin combination in a range of dose levels (Fig. 9A), while the treatment showed no significant toxicity (animal movement/behavior, fur shining, diarrhea, etc.) to animals including body weight loss (Fig. 9B). We further investigated whether the high in vivo anti-MPM tumor efficacy could be reflected at the level of molecular markers for tumor cell apoptosis, DNA damage and proliferation inhibition. Consistent with the in vitro data shown in Fig. 7AB, our IHC studies found the increased expression of active caspase-3 and γH2AX and decreased Ki67-positive cells in the tumor tissue (Fig. 10) as well as decreased antiapoptotic proteins, survivin and Bcl-XL (Supplemental Figure S7). The superior in vivo antitumor efficacy of FL496 compared to the FL496 + pemetrexed + cisplatin combination was further supported by the molecular profiles of the protein markers analyzed in our study (Fig. 10, Supplemental Figure S7). Lastly, we noticed that after FL496 treatment, while survivin positive cells significantly reduced, some large cells in the FL496-treated tumors showed extremely high survivin expression at the 4-week timepoint (Supplemental Figure S7A). We speculate that these large cells with high survivin expression could be some type of immune cells like M1 macrophages. However, this is an area that needs further investigation.

Pemetrexed-cisplatin combination together with the use of folic acid and vitamin B [] This 12 is not a citation but vitamin B12 is currently the best known treatment option to reduce toxicity in MPM patients [43–45]. Another possibility, based on the cisplatin + pemetrexed + FL496 treatment outcome, is the possible use of a low dose of FL496 as a weekly maintenance treatment after the initial treatment of MPM patients with cisplatin and pemetrexed. This is also an area for further exploration, which may obtain unexpected results showing greater anti-MPM efficacy with low toxicity in comparison with cisplatin + pemetrexed + FL496.

Additionally, from the results shown in Fig. 9A, it seems that the MPM tumor mouse group treated with 15 pem + 1.5 Cis (1/2 maximum tolerated dose/MTD) and the tumor mouse group treated with 30 pem + 3 Cis (MTD) exhibited similar anti-MPM tumor efficacy. However, the initial tumor size for the 15 pem + 1.5 Cis mouse group was only about 200 mm^3^, whereas the initial tumor size for the 30 pem + 3 Cis mouse group was almost 400 mm^3^. Considering this, the reality is that 30 pem + 3 Cis exhibited better anti-MPM activity than 15 pem + 1.5 Cis. After treatment, the tumor growth curve from the 15 pem + 1.5 Cis mouse group caught up to the tumor growth curve from the 30 pem + 3 Cis mouse group (Fig. 9A), which indicates that 30 pem + 3 Cis is more effective in inhibiting tumor growth than 15 pem + 1.5 Cis, though without adding a low dose of FL496, both treatments only exhibited a minimal delay in tumor growth (Fig. 9A).

In summary, the combination of cisplatin and pemetrexed is the current standard first-line chemotherapy for MPM. The cisplatin/ pemetrexed combined mechanism of action (MOA) targets DNA replication and nucleotide biosynthesis, leading to enhanced cancer cell death. Specifically, the platinum-based cisplatin induces intra- and inter-strand DNA crosslinks (DNA damage), causes replication fork stalling, leads to cell cycle arrest, apoptosis and the production of reactive oxygen species (ROS) to induce cytotoxicity. In contrast, the multitargeted antifolate agent pemetrexed inhibits multiple nucleotide biosynthesis enzymes (e.g., thymidylate synthase/TS, dihydrofolate reductase/DHFR). Hence, pemetrexed can deplete cancer DNA and RNA precursors, thus impairing DNA replication and repair. Therefore, the combination of cisplatin and pemetrexed could block cancer DNA/RNA synthesis and cause DNA damage and synergestic cancer cell death/apoptosis. Although the exact MOA of FL496 remains to be fully elucidated, our studies have helped define a clear framework for understanding its MOA. We have confirmed that FL496 uses both p53-dependent and p53-independent MOA to induce MPM cell growth inhibition and apoptosis induction. This is likely through inducing MPM cell senescence, DNA damage (e.g., its induction of the DNA damage marker γH2AX) and the inhibition of antiapoptotic proteins (e.g., survivin, Mcl-1, Bcl-2 and Bcl-XL, etc.). Furthermore, like FL118 [49], inhibition of DDX5 by FL496 is likely at least partially involved in FL496-induced DNA damage. Additionally, DDX5 is known to play a role in cancer metabolic promotion and immune suppression [49]. Therefore, the role of FL118 and FL496 in controlling cancer metabolic effects and immune suppression release would be an interesting research area and require further investigation in the coming years.

A final note is that we used additional non-mesothelioma cell lines in this report. This includes [] This 1 and the below 2 and 3 are not citation but three points, which should be marked in the format of (1), (2), (3) the use of HPV-negative cervical cancer HT-3 cell lines for our inital screening to identify FL7N-1 as the best compound for comparison with FL496 among the three heterocyclic ring FL118 position 7 derivatives; [] the use of the rare cancer osteosarcoma U2OS cell lines to demonstrate the effect of FL496 on the induction of DNA damage marker γH2AX similar to those for FL496 to induce γH2AX in mesothelioma cell models; and [] the use of 4 additional pediatric osteosarcoma PDX tumor-established cell lines to functionally demonstrate the high efficacy of FL496 to inhibit osteosarcom cell growth (Fig. 6). These findings from non-MPM cell models further strengthened the data obtained from the MPM cell and tumor model, and at least partially alleviated the limitation of our studies using two mesothelioma cell models for the in vitro and in vivo studies.

## Conclusions

**Fig. 11:**
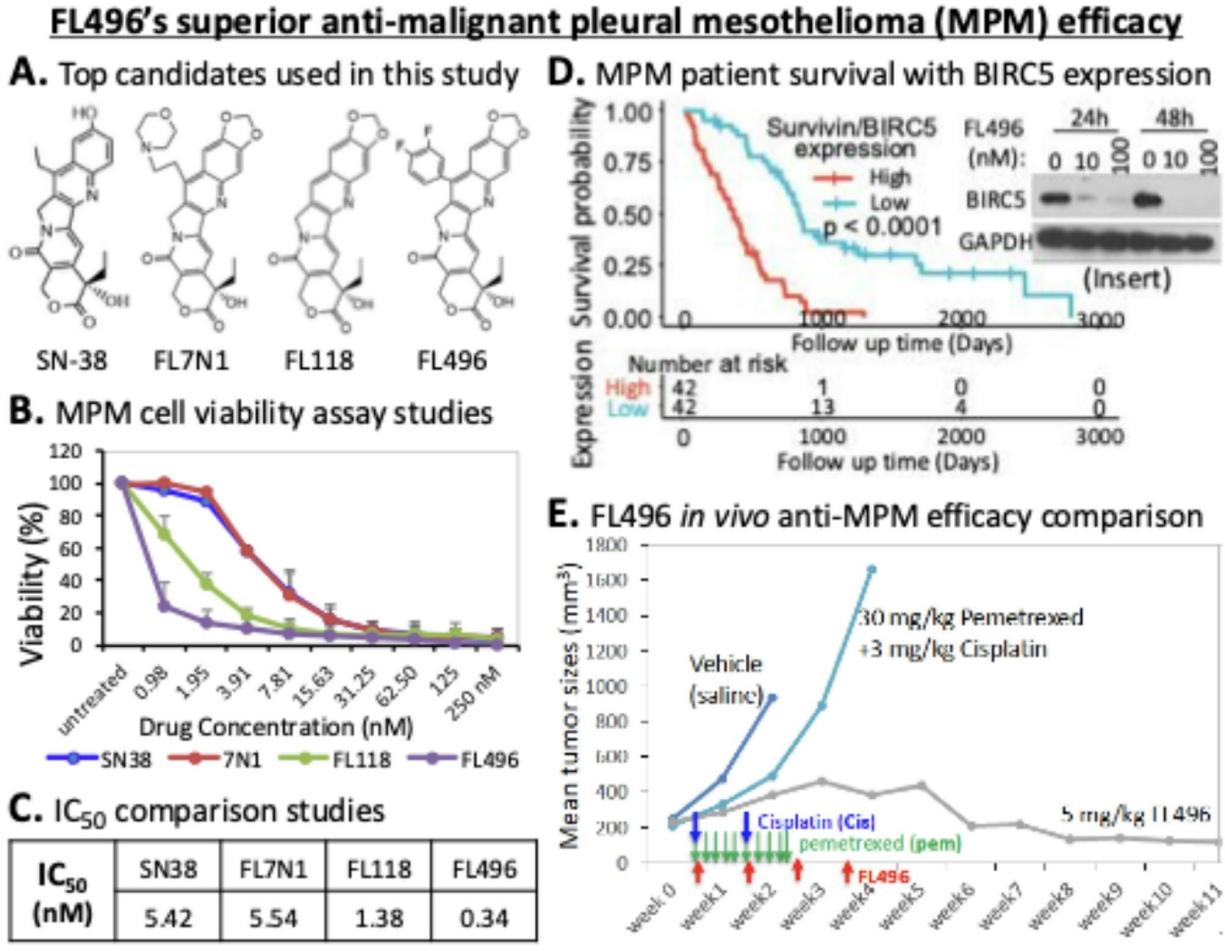
Summary of representative results obtained in this study. FL496 exhibits anti-malignant pleural mesothelioma (MPM) efficacy strikingly superior to the antitumor efficacy resulting from the treatment with pemetrexed-cisplatin combination which is the standard first-line chemotherapy in the clinic for MPM treatent over the past 20 + years in the clinic practice for MPM treatment

Based on the key results derived from our in vitro and in vivo studies summarized in Fig. 11, we conclude that our identified FL118-derivative small molecule compound FL496 holds promise for development as a monotherapeutic agent for the effective treatment of MPM, with potential applicability to osteosarcoma as well. Therefore, FL496 is worthy of continued development toward clinical trials in MPM patients and osteosarcoma patients.

## Supplementary Information

Below is the link to the electronic supplementary material.

Supplementary Material 1

## Data Availability

Data that are necessary to interpret, verify and extend the research in the article transparent to readers have been provided in figures and supplemental materials. No more data available.

## Abbreviations

β-gal: β-Galactosidase
BIRC5: Baculoviral IAP repeat containing 5
bMPM: Biphasic malignant pleural mesothelioma
BSA: Bovine serum albumin
CRC: Colorectal cancer
eMPM: Epithelioid malignant pleural mesothelioma
FDA: Food and drug administration
FH: Fumarate hydratase
FHpRCC: FH deficiency-induced type-2 papillary renal cell carcinoma
h: Hour(s)
HPβCD: 2-Hydroxypropyl-β-cyclodextrin
IACUC: Institutional animal care and use committee
IAP: Inhibitor of apoptosis
IF: Immunofluorescence
ip: Intraperitoneal
KO: Knockout
MOA: Mechanism of action
MPM: Malignant pleural mesothelioma
Nrf2: NF-E2 p45-related factor 2
PDAC: Pancreatic ductal adenocarcinoma
PDC: Pyridinium dichromate
PDX: Patient-derived xenograft
RCC: Renal cell carcinoma
SCID: Severe combined immunodeficiency
sMPM: Sarcomatoid malignant pleural mesothelioma
