# Supplementary material for: FL496, an FL118-derived small molecule, induces growth inhibition, senescence, and apoptosis of malignant pleural mesothelioma (MPM) cells, and exhibits anti-MPM tumor efficacy strikingly superior to the pemetrexed-cisplatin combination
Source: J Exp Clin Cancer Res. 2025 Oct 21;44:293. doi: 10.1186/s13046-025-03547-9 (PMC12538866; doi:10.1186/s13046-025-03547-9)
Supplement: Supplementary file 1 — Supplementary Material 1 [file 13046_2025_3547_MOESM1_ESM.pdf]

Supplemental Material Summary

Supplemental Figures S1 to S7 …………………………………….. Pages 2 - 8

Supplemental Tables S1 to S4……………………………………… Pages 9 – 12

Supplemental Figures:

(A)

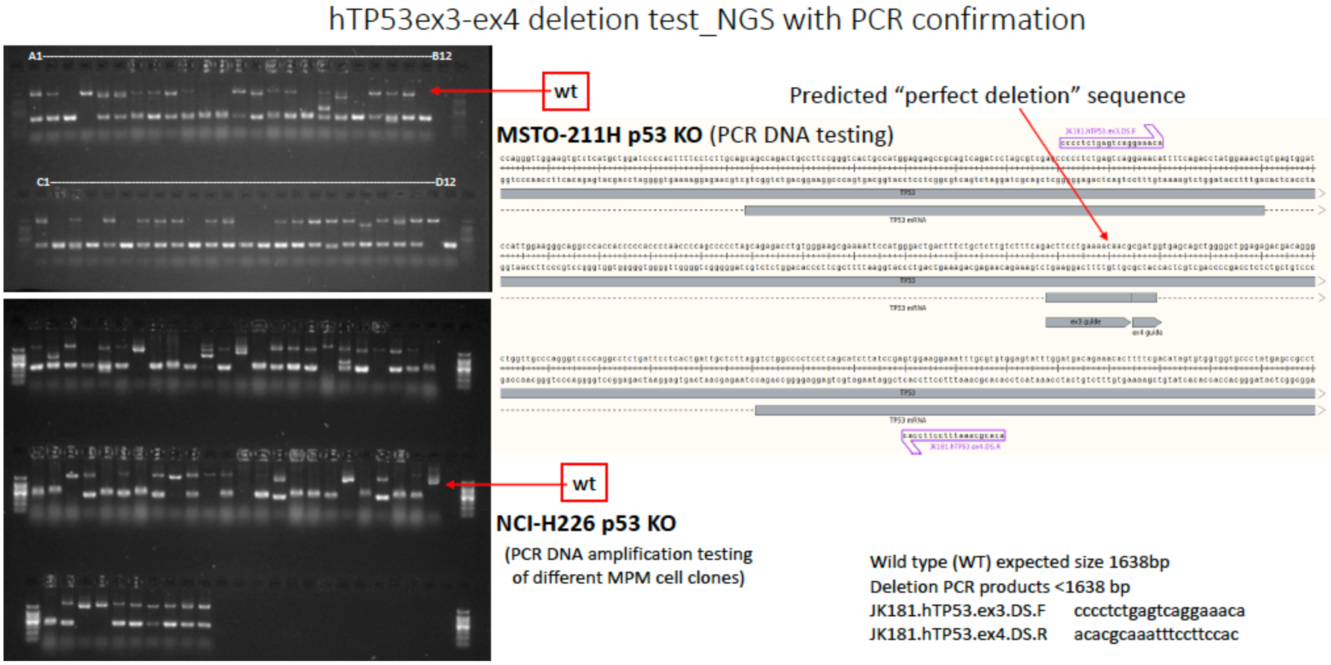

(B)

NGS Summary Analysis

The Excel spread sheet will give details about the various indels found in each cell clone sample based on length compared to the WT sequence. When looking for indels, the columns titled "#1-Indel," "#2-Indel," etc. represent the length of the NHEJ event if any occurred (i.e., a -1 represents a 1bp deletion; a 10 represents a 10bp insertion; a 0 represents WT or a point mutation since a point mutation would be the same length as a WT read). The "Reads(%)" column associated with each indel column gives the number of reads and percent of the total amount of reads that align to that specific indel.

The specific sequence for the 12 most abundant reads for each sample are found in the results_counter file. At the end of the line in the results_counter file that contains the name of the well, the indel length, and the count for each indel type is listed in brackets (i.e. [(0, 4523), (1, 342), (-2, 23)]). The following sequences for each well are broken down into the various forms of each indel.

Supplemental Figure S1. (**A**) Human *TP53* (p53 gene) exon 3-exon 4 deletion PCRT confirmation using next generation sequencing (NSG). (**B**) NSG summary analysis. **wt**: wild type

(A)

Results_counter Example

Internal Project ID
The_Seq_start: "sequence along amplicon where our query begins"
The_Seq_end: "sequence along amplicon where our query ends"
Test_Sequences:
g4: "sequence of WT gRNA"

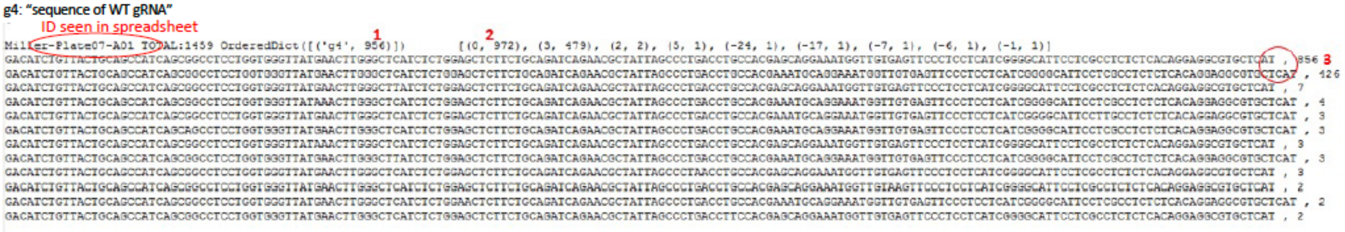

1) Reads that exactly align to query sequence "g4" as listed at the top left (WT gRNA sequence)

2) Various indels sizes with associated read counts

3) Number of reads for that specific sequence. You can see that the sequences next to item 3 appear to be of different lengths. If you align those sequences, you can determine the specific indel that is present.

*Note that the sequences with ≤1% coverage are background reads from PCR error. Even though these are erroneous sequences, they still add to the count for the specific indel size that they represent. This is why you will see totals in item 2 that differ from read counts at the end of sequence lines.

(B)

Summary file example

Number of reads per well

Modification

Wild type reads

Indel, reads count and % of reads

SNP=ratio of 5' and 3' reads, QC control

Name	Sample	Total	Q670*	sp4	#1-Indel	#1-Reads(%)	#2-Indel	#2-Reads(%)	#3-Indel	#3-Reads(%)	#4-Indel	#4-Reads(%)	#5-Indel	#5-Reads(%)	#6-Indel	#6-Reads(%)	#7-Indel	#7-Reads(%)	#8-Indel	#8-Reads(%)	SNP_test	raw_wt_counter
GEIC-Plate13-A01		1759	0 (0.0%)	0 (0.0%)	-3	558 (31.7%)	1	411 (23.4%)	0	390 (22.2%)	-11	364 (20.7%)	-4	15 (0.9%)	-1	11 (0.6%)	-12	6 (0.3%)	-5	2 (0.1%)	1	0
GEIC-Plate13-A02		6765	0 (0.0%)	0 (0.0%)	-3	3318 (49.0%)	-8	3250 (48.0%)	-9	102 (1.5%)	-4	86 (1.3%)	-10	3 (0.0%)	-5	2 (0.0%)	-2	2 (0.0%)	1	1 (0.0%)	1.02	0
GEIC-Plate13-A03		1734	0 (0.0%)	0 (0.0%)	-3	870 (50.2%)	1	815 (47.0%)	-4	26 (1.5%)	0	17 (1.0%)	-8	2 (0.1%)	-5	2 (0.1%)	-87	1 (0.1%)	-1	1 (0.1%)	1.01	0
GEIC-Plate13-A04		1354	0 (0.0%)	0 (0.0%)	-3	611 (45.1%)	-11	454 (33.5%)	-141	254 (18.8%)	-4	19 (1.4%)	-12	13 (1.0%)	-143	2 (0.1%)	-142	1 (0.1%)	NA		1.03	0
GEIC-Plate13-A05		2042	1 (0.0%)	0 (0.0%)	0	932 (45.6%)	-11	882 (43.2%)	-10	92 (4.5%)	1	79 (3.9%)	-12	27 (1.3%)	-1	26 (1.3%)	-13	2 (0.1%)	-2	2 (0.1%)	1.02	1 (1.0)

Supplemental Figure S2. (**A**) The counter example of Human *TP53* (p53 gene) exon 3-exon 4 deletion results. (**B**) The Human *TP53* (p53 gene) exon 3-exon 4 deletion result summary file example

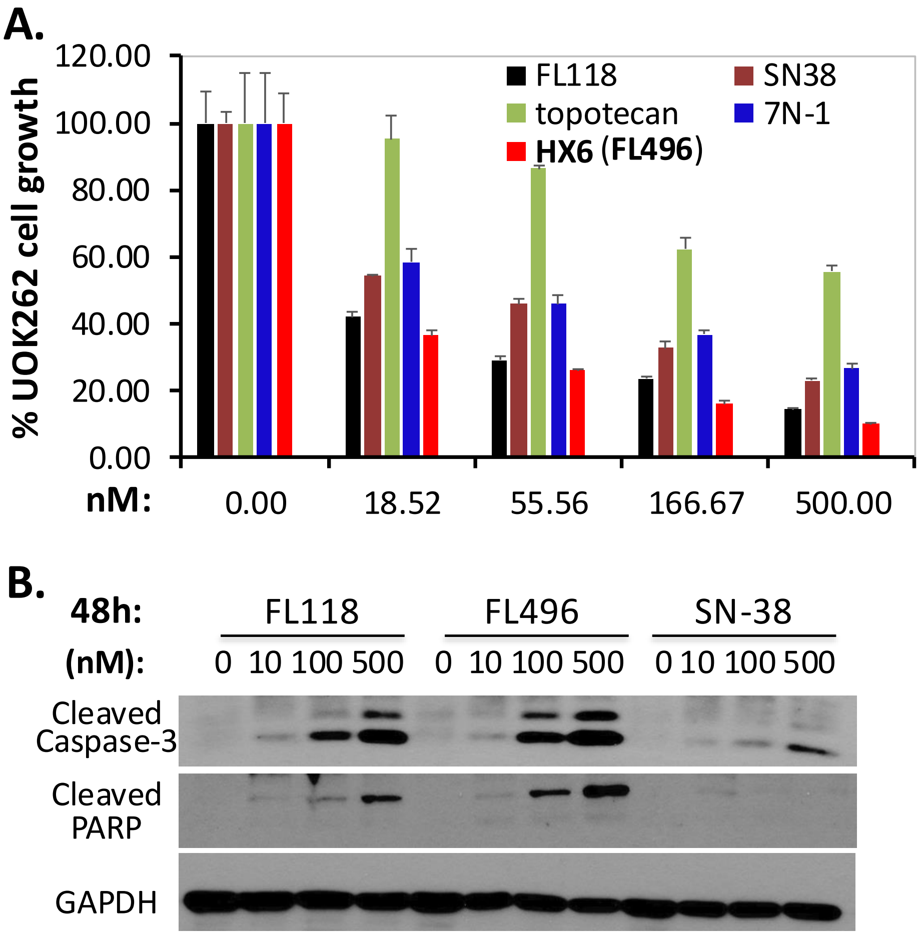

Supplemental Figure S3. Comparison of the relative potency of FL118, FL7N1 and FL496 in the fumarate hydratase (FH) genetic defect papillary renal cell carcinoma (FHpRCC) cells. (**A**) Inhibition of the FHpRCC patient-derived UOK262 cells by FL118 FL7N1 and FL496 in parallel with the structure-relevant positive control drugs, SN38 and topotecan. UOK262 cells were seeded in 96-well plates and treated with and without FL118, FL7N1, FL496, SN-38 and topotecan for 72 h with a series of drug concentrations as shown. Cell growh/viability was then determined using MTT assay. The data is the mean + SD from three independent assays. (**B**) Comparison of the induction of UOK262 cell apoptosis by FL118, FL496 and SN-38. Subconfluent UOK262 cells in 6-well plates were treated with FL118, FL496 and SN-38 at the concentration of 0 nM (control), 10 nM, 100 nM and 500 nM as shown for 48h. Cells were then lysed for Western blot analyses with antibodies for cleaved caspase-3, cleaved PARP and GAPDH. The expression GAPDH is the internal protein loading control. Of note, cleaved/active caspose-3 and cleaved PARP are the hallmark of cell apoptosis. Of note, the data shown in A is an expending update from a previously reported data (Figure 19A; Li, et al. J Exp Clin Cancer Res 40:254, 2021)

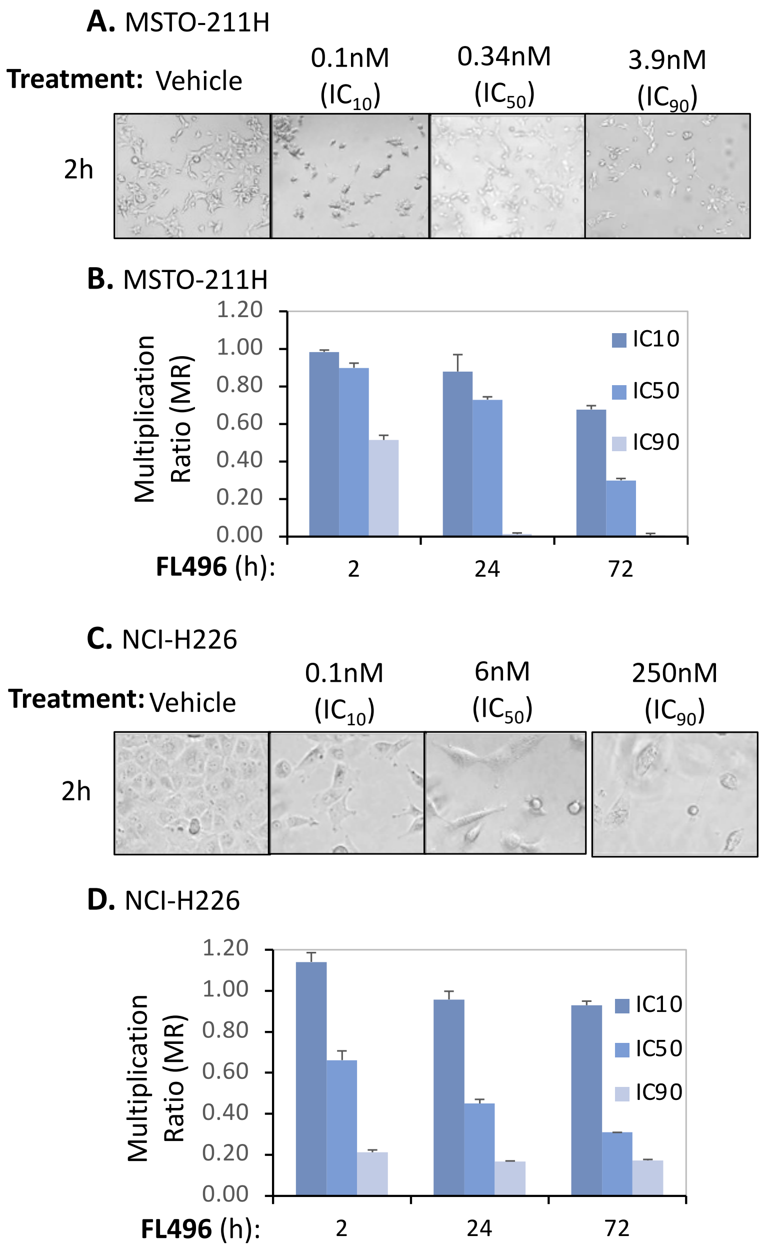

Supplemental Figure S4. FL496 alters phenotypic characteristics and replicative capacity of MPM cells in culture: (**A**) FL496 treatment can rapidly change the MSTO-211H MPM cell morphology. Representative morphologic change profile images of these cells with 2h FL496 treatment were taken using a light microscope after 72h removal of FL496. (**B**) FL496 modulates the proliferative capacity of MSTO-211H MPM cells calculated as the multiplication ratio of the cells. Data were diagramed in histogram from multiple triplicate image calculation. Each bar is the mean + SD derived from multiple image calculation (n=3). (**C**) FL496 treatment can rapidly change the NCI-H226 MPM cell morphology. Representative morphological change profile images of these cells with 2h FL496 treatment were taken using a light microscope after 72h removal of FL496. (**D**) FL496 modulates the proliferative capacity of NCI-H226 MPM cells calculated as the multiplication ratio of the cells. Data were diagramed in histogram from multiple triplicate image calculation. Each bar is the mean + SD derived from multiple image calculation (n=3).

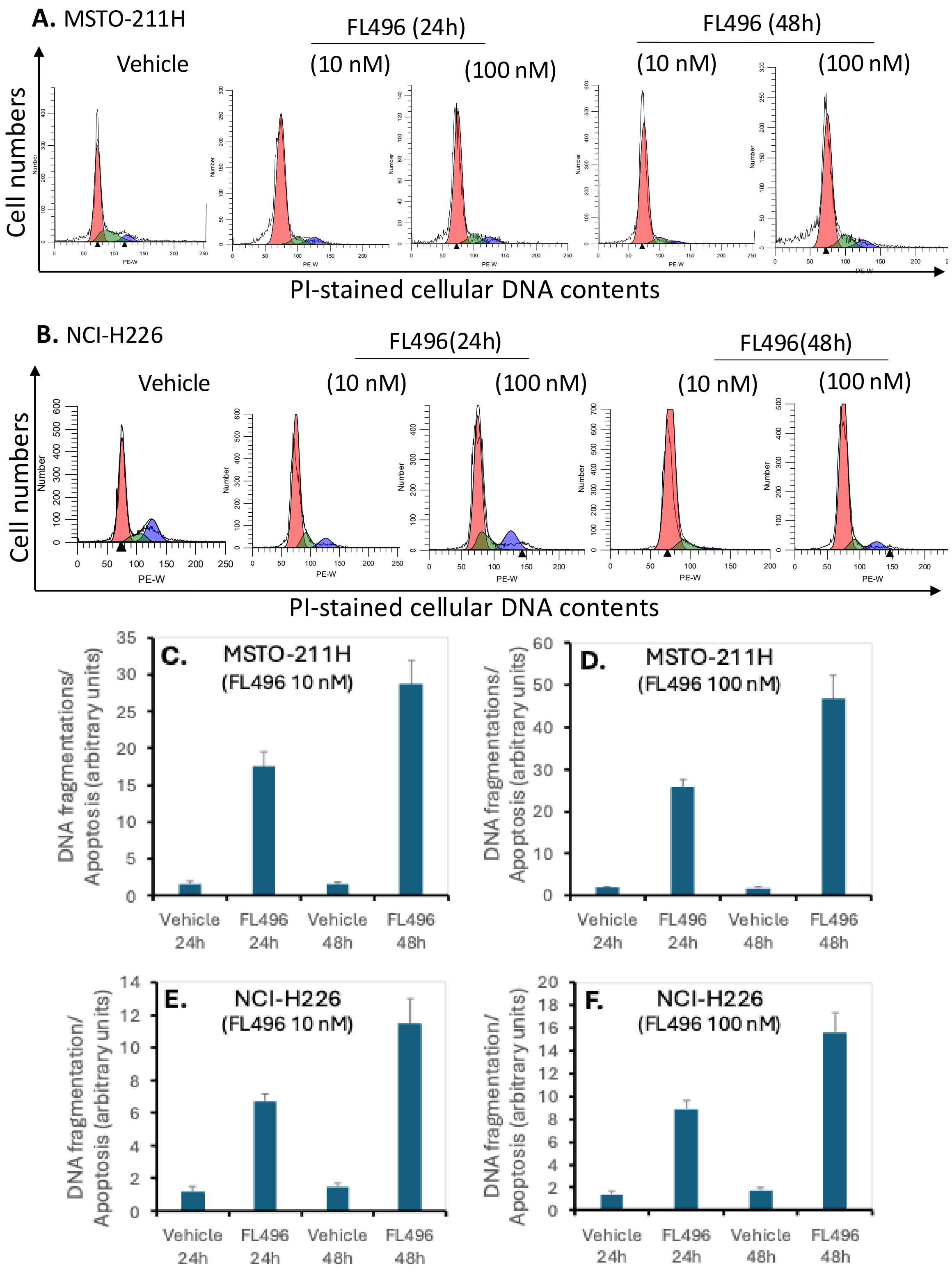

Supplemental Figure S5. FL496 treatment induces living MPM cells arrested in G1 phase. (**A**, **B**) Representative profiles of cell cycle distribution for MSTO-211H (A), and NCI-H226 (B) cells are shown after FL496 treatment. Alternatively, the MPM cell DNA fragmentation (apoptosis) were determined

using a DNA fragmentation cell death ELISA assay kit. Results derived from MSTO-211H cells treated with FL496 at 10 nM (**C**) and 100 nM (**D**) for 24h and 48h were shown. Results derived from NCI-H226 cells treated with FL496 at 10 nM (**E**) and 100 nM (**F**) for 24h and 48h were shown.

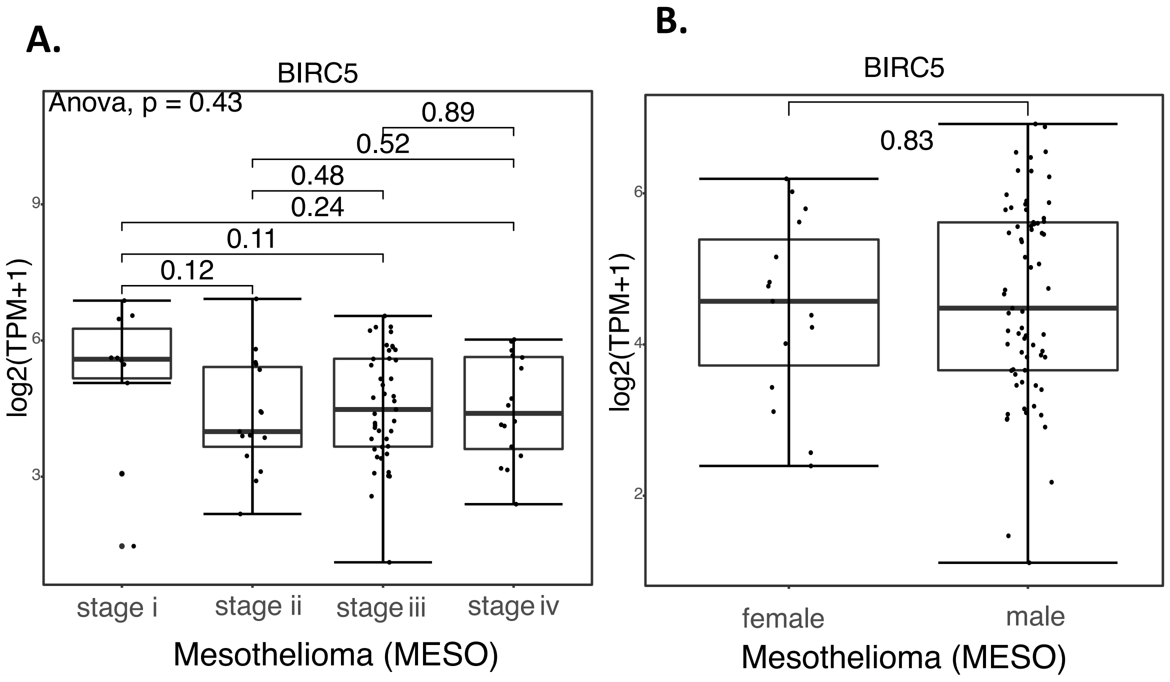

Supplemental Figure S6. Survivin (BIRC5) expression comparison among various stages of MPM (stage i-iv, **A**) or among female and male patients (**B**) from the TCGA mesothelioma (MESO) dataset in the boxplot plot format. BIRC5/survivin expression was presented in the log2 (TPM+ 1) scale format.

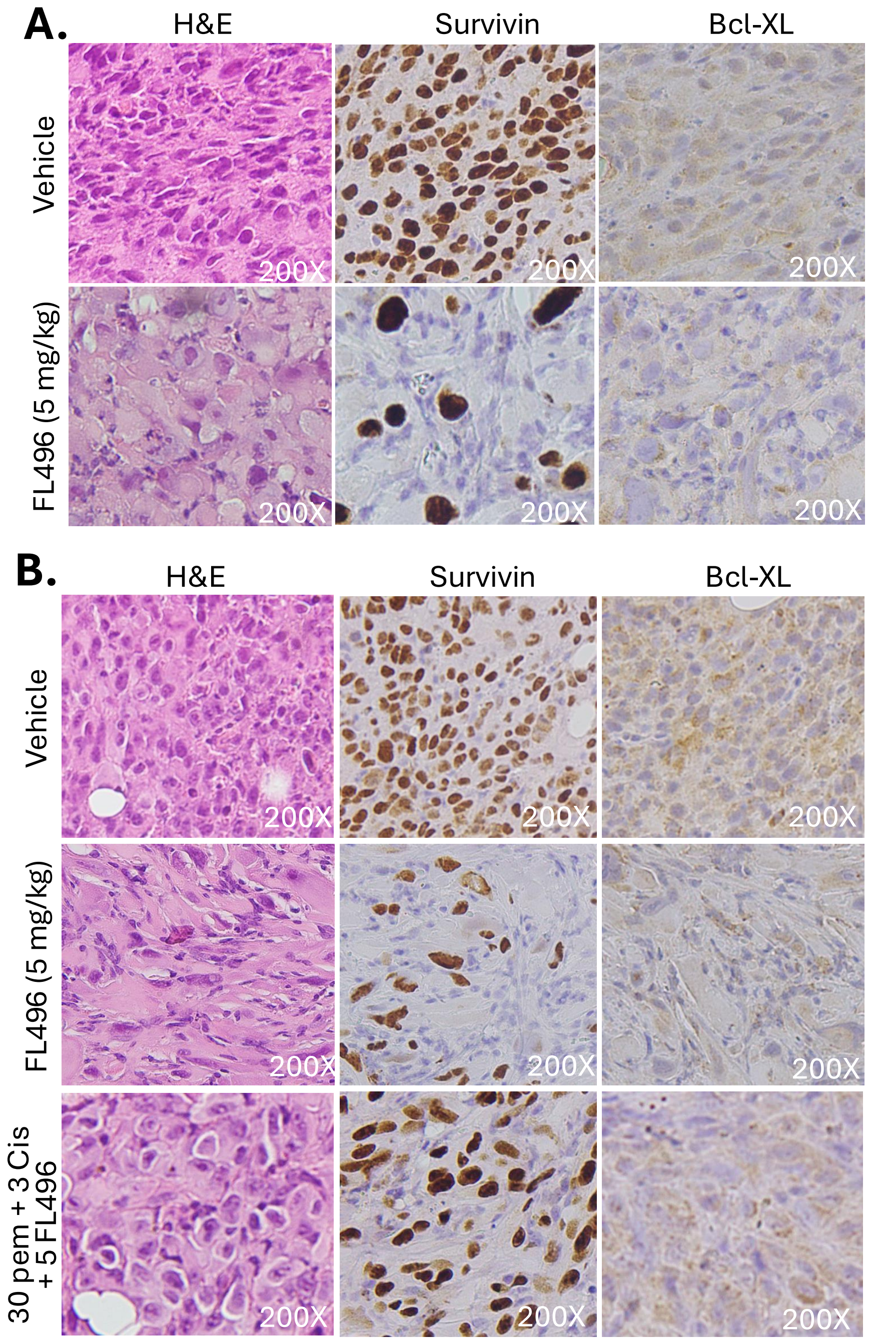

Supplemental Figure S7. FL496 decreased the expression of the antiapoptotic proteins, survivin and Bcl-XL in FL496-treated mouse-derived tumors. Representative images were shown for the 4-week timepoint (**A**) and 8-week time point (**B**).

Supplemental Tables:

Supplemental Table S1: Antibodies used in this study report

Antibody	species	Provider	Catalog number
β-actin	Mouse	CST*	4967
BCL-2	Rabbit	abcam	ab182858
BCL-XL	Mouse	SC	sc-8392
cIAP-2	Rabbit	CST	3130
Caspase-3 (D3R6Y)	Rabbit	CST	14220
Cleaved caspase 3	Rabbit	CST	D3R6Y
GAPDH	Mouse	CST	2118
Mcl-1	Rabbit	CST	4572s
MDM2	Mouse	proteintech	66511-1-Ig
MDMX	Rabbit	proteintech	17914-1-AP
N-Myc (B8.4.B)	Mouse	SC	sc-53993
p21	Rabbit	CST	2947
p53 (DO-1)	Mouse	SC	126
PARP	Rabbit	CST	9542
Phospho-p53 (S15)	Rabbit	CST	9284
Phospho-Rb (E-10)	Mouse	SC	271930
PUMA	Rabbit	CST	4976S
Rb	Mouse	SC	sc-102
Survivin	Rabbit	CST	2808
XIAP	Rabbit	CST	2042S
α-tubulin	Mouse	proteintech	66031-1-Ig
γH2Ax (Ser139)	Mouse	Millipore	05-636
γH2Ax (Ser139)	Rabbit	CST	9718
Phospho –ATM (Ser1981)	Rabbit	CST	5883
ATM (D2E2)	Rabbit	CST	2873
DDX5	Mouse hybridoma	R&D Systems	MAB6370
Ki67	Rabbit	abcam	Ab15580

* CST: Cell Signaling Technology

Supplemental Table S2: JK181_hTP53.ex3 summary.xlsx

Name	Sample	Total	guide3_wt	#1-Indel	#1-Reads(%)	#2-Indel	#2-Reads(%)	#3-Indel	#3-Reads(%)	#4-Indel	#4-Reads(%)
GEIC-Plate29-A01	MSTO-211H p53 KO	343	325 (94.8%)	-16	337 (98.3%)	-17	5 (1.5%)	-15	1 (0.3%)	NA	
GEIC-Plate29-A02	MSTO-211H p53 KO	1024	0 (0.0%)	-33	1016 (99.2%)	-34	6 (0.6%)	-35	2 (0.2%)	NA	
GEIC-Plate29-A03	MSTO-211H p53 KO										
GEIC-Plate29-A04	MSTO-211H p53 KO	1556	0 (0.0%)	-40	1547 (99.4%)	-41	9 (0.6%)	NA		NA	
GEIC-Plate29-A05	MSTO-211H p53 KO										
GEIC-Plate29-A06	MSTO-211H p53 KO										
GEIC-Plate29-A07	MSTO-211H p53 KO	669	10 (1.5%)	-23	654 (97.8%)	-16	11 (1.6%)	-24	4 (0.6%)	NA	
GEIC-Plate29-A08	MSTO-211H p53 KO										
GEIC-Plate29-A09	MSTO-211H p53 KO										
GEIC-Plate29-A10	MSTO-211H p53 KO	959	2 (0.2%)	-20	948 (98.9%)	-21	8 (0.8%)	-16	2 (0.2%)	-19	1 (0.1%)
GEIC-Plate29-A11	MSTO-211H p53 KO										
GEIC-Plate29-A12	MSTO-211H p53 KO										
GEIC-Plate29-B01	MSTO-211H p53 KO	1805	880 (48.8%)	-16	907 (50.2%)	-15	891 (49.4%)	-17	7 (0.4%)	NA	
GEIC-Plate29-B02	MSTO-211H p53 KO	1467	1427 (97.3%)	-16	1448 (98.7%)	-17	17 (1.2%)	0	1 (0.1%)	-18	1 (0.1%)
GEIC-Plate29-B03	MSTO-211H p53 KO	1666	1602 (96.2%)	-16	1632 (98.0%)	-40	17 (1.0%)	-17	14 (0.8%)	0	2 (0.1%)
GEIC-Plate29-B04	MSTO-211H p53 KO	1971	1 (0.1%)	-32	1000 (50.7%)	-20	959 (48.7%)	-21	7 (0.4%)	-33	4 (0.2%)
GEIC-Plate29-B05	MSTO-211H p53 KO	19	2 (10.5%)	-18	16 (84.2%)	-16	1 (5.3%)	0	1 (5.3%)	-17	1 (5.3%)
GEIC-Plate29-B06	MSTO-211H p53 KO	1985	1942 (97.8%)	-16	1969 (99.2%)	-17	14 (0.7%)	-15	1 (0.1%)	-13	1 (0.1%)
GEIC-Plate29-B07	MSTO-211H p53 KO	479	347 (72.4%)	-16	360 (75.2%)	-42	117 (24.4%)	-17	1 (0.2%)	-43	1 (0.2%)
GEIC-Plate29-B08	MSTO-211H p53 KO										
GEIC-Plate29-B09	MSTO-211H p53 KO	1592	1550 (97.4%)	-16	1574 (98.9%)	-17	18 (1.1%)	NA		NA	
GEIC-Plate29-B10	MSTO-211H p53 KO	1554	1505 (96.8%)	-16	1541 (99.2%)	-17	11 (0.7%)	-15	1 (0.1%)	-38	1 (0.1%)
GEIC-Plate29-B11	MSTO-211H p53 KO	1668	4 (0.2%)	-49	829 (49.7%)	-43	822 (49.3%)	-16	5 (0.3%)	-31	4 (0.2%)
GEIC-Plate29-B12	MSTO-211H p53 KO	34	24 (70.6%)	-16	15 (44.1%)	0	11 (32.4%)	-18	4 (11.8%)	-22	4 (11.8%)
GEIC-Plate29-C01	MSTO-211H p53 KO	1578	0 (0.0%)	-32	1566 (99.2%)	-33	12 (0.8%)	NA		NA	
GEIC-Plate29-C02	MSTO-211H p53 KO										
GEIC-Plate29-C03	MSTO-211H p53 KO	1400	1 (0.1%)	-24	1386 (99.0%)	-25	11 (0.8%)	-26	2 (0.1%)	0	1 (0.1%)
GEIC-Plate29-C04	MSTO-211H p53 KO										
GEIC-Plate29-C05	MSTO-211H p53 KO	1778	1730 (97.3%)	-16	1762 (99.1%)	-17	15 (0.8%)	-18	1 (0.1%)	NA	
GEIC-Plate29-C06	MSTO-211H p53 KO										
GEIC-Plate29-C07	MSTO-211H p53 KO										
GEIC-Plate29-C08	MSTO-211H p53 KO										
GEIC-Plate29-C09	MSTO-211H p53 KO										
GEIC-Plate29-C10	MSTO-211H p53 KO	1597	1421 (89.0%)	-16	1453 (91.0%)	-31	118 (7.4%)	-17	23 (1.4%)	-32	3 (0.2%)
GEIC-Plate29-C11	MSTO-211H p53 KO	1815	1 (0.1%)	-38	1801 (99.2%)	-39	12 (0.7%)	-16	1 (0.1%)	0	1 (0.1%)
GEIC-Plate29-C12	MSTO-211H p53 KO										
GEIC-Plate29-D01	MSTO-211H p53 KO										
GEIC-Plate29-D02	MSTO-211H p53 KO	1353	0 (0.0%)	-15	1342 (99.2%)	-16	10 (0.7%)	-17	1 (0.1%)	NA	
GEIC-Plate29-D03	MSTO-211H p53 KO	1227	0 (0.0%)	-17	1219 (99.3%)	-18	8 (0.7%)	NA		NA	
GEIC-Plate29-D04	MSTO-211H p53 KO										
GEIC-Plate29-D05	MSTO-211H p53 KO										
GEIC-Plate29-D06	MSTO-211H p53 KO	1089	1 (0.1%)	-18	1083 (99.4%)	-19	4 (0.4%)	-16	1 (0.1%)	-42	1 (0.1%)
GEIC-Plate29-D07	MSTO-211H p53 KO										
GEIC-Plate29-D08	MSTO-211H p53 KO	1720	1 (0.1%)	-30	1702 (99.0%)	-31	15 (0.9%)	-29	1 (0.1%)	-32	1 (0.1%)
GEIC-Plate29-D09	MSTO-211H p53 KO	1940	0 (0.0%)	-17	1921 (99.0%)	-18	19 (1.0%)	NA		NA	
GEIC-Plate29-D10	MSTO-211H p53 KO	903	522 (57.8%)	-16	531 (58.8%)	-40	362 (40.1%)	-17	6 (0.7%)	-41	3 (0.3%)
GEIC-Plate29-D11	MSTO-211H p53 KO	1373	1 (0.1%)	-31	703 (51.2%)	-17	650 (47.3%)	-18	11 (0.8%)	-32	5 (0.4%)

Supplemental Table S3: JK181_hTP53.ex3-4 deletion summary.xlsx

Name	Sample	Total	perfect deletion	guide4	guide3	#1-Indel	#1-Reads(%)	#2-Indel	#2-Reads(%)	#3-Indel	#3-Reads(%)	#4-Indel	#4-Reads(%)
GEIC-Plate31-A01	MSTO-211H p53 KO	619	0 (0.0%)	0 (0.0%)	0 (0.0%)	-2	614 (99.2%)	-3	4 (0.6%)	-1	1 (0.2%)	NA	
GEIC-Plate31-A02	MSTO-211H p53 KO	1626	809 (49.8%)	0 (0.0%)	0 (0.0%)	0	824 (50.7%)	-12	796 (49.0%)	-1	3 (0.2%)	-13	2 (0.1%)
GEIC-Plate31-A03	MSTO-211H p53 KO	1585	1541 (97.2%)	0 (0.0%)	0 (0.0%)	0	1574 (99.3%)	-1	11 (0.7%)	NA		NA	
GEIC-Plate31-A04	MSTO-211H p53 KO												
GEIC-Plate31-A05	MSTO-211H p53 KO	1189	1143 (96.1%)	0 (0.0%)	0 (0.0%)	0	1187 (99.8%)	-1	2 (0.2%)	NA		NA	
GEIC-Plate31-A06	MSTO-211H p53 KO	1092	1051 (96.2%)	0 (0.0%)	0 (0.0%)	0	1089 (99.7%)	-1	3 (0.3%)	NA		NA	
GEIC-Plate31-A07	MSTO-211H p53 KO	1150	966 (84.0%)	0 (0.0%)	0 (0.0%)	0	1005 (87.4%)	-42	141 (12.3%)	-1	4 (0.3%)	NA	
GEIC-Plate31-A08	MSTO-211H p53 KO	1263	1220 (96.6%)	0 (0.0%)	0 (0.0%)	0	1259 (99.7%)	-1	3 (0.2%)	-2	1 (0.1%)	NA	
GEIC-Plate31-A09	MSTO-211H p53 KO	1080	1046 (96.9%)	0 (0.0%)	0 (0.0%)	0	1076 (99.6%)	-1	4 (0.4%)	NA		NA	
GEIC-Plate31-A10	MSTO-211H p53 KO	953	0 (0.0%)	0 (0.0%)	0 (0.0%)	-17	948 (99.5%)	-18	4 (0.4%)	-60	1 (0.1%)	NA	
GEIC-Plate31-A11	MSTO-211H p53 KO	1193	1171 (98.2%)	0 (0.0%)	0 (0.0%)	0	1191 (99.8%)	-1	2 (0.2%)	NA		NA	
GEIC-Plate31-A12	MSTO-211H p53 KO	125	63 (50.4%)	0 (0.0%)	0 (0.0%)	0	63 (50.4%)	-2	61 (48.8%)	-1	1 (0.8%)	NA	
GEIC-Plate31-B01	MSTO-211H p53 KO	580	362 (62.4%)	0 (0.0%)	0 (0.0%)	0	375 (64.7%)	-2	199 (34.3%)	-1	4 (0.7%)	-60	1 (0.2%)
GEIC-Plate31-B02	MSTO-211H p53 KO	1334	20 (1.5%)	0 (0.0%)	0 (0.0%)	-10	1304 (97.8%)	0	20 (1.5%)	-11	7 (0.5%)	-25	2 (0.1%)
GEIC-Plate31-B03	MSTO-211H p53 KO	783	13 (1.7%)	0 (0.0%)	0 (0.0%)	-7	758 (96.8%)	0	16 (2.0%)	-18	6 (0.8%)	-8	3 (0.4%)
GEIC-Plate31-B04	MSTO-211H p53 KO	1468	1362 (92.8%)	0 (0.0%)	0 (0.0%)	0	1399 (95.3%)	-84	63 (4.3%)	-1	6 (0.4%)	NA	
GEIC-Plate31-B05	MSTO-211H p53 KO	1050	518 (49.3%)	0 (0.0%)	0 (0.0%)	0	526 (50.1%)	-2	519 (49.4%)	-1	4 (0.4%)	-3	1 (0.1%)
GEIC-Plate31-B06	MSTO-211H p53 KO	1213	0 (0.0%)	0 (0.0%)	0 (0.0%)	-23	1199 (98.8%)	150	6 (0.5%)	147	3 (0.2%)	-24	1 (0.1%)
GEIC-Plate31-B07	MSTO-211H p53 KO	1057	187 (17.7%)	0 (0.0%)	0 (0.0%)	-31	439 (41.5%)	-37	422 (39.9%)	0	191 (18.1%)	-32	3 (0.3%)
GEIC-Plate31-B08	MSTO-211H p53 KO	1400	489 (34.9%)	0 (0.0%)	0 (0.0%)	-12	532 (38.0%)	0	496 (35.4%)	-2	370 (26.4%)	-3	1 (0.1%)
GEIC-Plate31-B09	MSTO-211H p53 KO	1020	968 (94.9%)	0 (0.0%)	0 (0.0%)	0	1015 (99.5%)	-1	4 (0.4%)	-2	1 (0.1%)	NA	
GEIC-Plate31-B10	MSTO-211H p53 KO	906	0 (0.0%)	0 (0.0%)	0 (0.0%)	-2	517 (57.1%)	-74	388 (42.8%)	-3	1 (0.1%)	NA	
GEIC-Plate31-B11	MSTO-211H p53 KO	1549	1499 (96.8%)	0 (0.0%)	0 (0.0%)	0	1543 (99.6%)	-1	5 (0.3%)	-26	1 (0.1%)	NA	
GEIC-Plate31-B12	MSTO-211H p53 KO	1481	1434 (96.8%)	0 (0.0%)	0 (0.0%)	0	1478 (99.8%)	-1	3 (0.2%)	NA		NA	
GEIC-Plate31-C01	MSTO-211H p53 KO	1338	1264 (94.5%)	0 (0.0%)	0 (0.0%)	0	1303 (97.4%)	-37	12 (0.9%)	-45	9 (0.7%)	-1	5 (0.4%)
GEIC-Plate31-C02	MSTO-211H p53 KO	1559	437 (28.0%)	0 (0.0%)	0 (0.0%)	0	446 (28.6%)	-26	437 (28.0%)	-35	419 (26.9%)	-15	255 (16.4%)
GEIC-Plate31-C03	MSTO-211H p53 KO	1477	3 (0.2%)	0 (0.0%)	0 (0.0%)	2	800 (54.2%)	-50	669 (45.3%)	0	3 (0.2%)	1	3 (0.2%)
GEIC-Plate31-C04	MSTO-211H p53 KO	1452	1419 (97.7%)	0 (0.0%)	0 (0.0%)	0	1448 (99.7%)	-1	3 (0.2%)	-50	1 (0.1%)	NA	
GEIC-Plate31-C05	MSTO-211H p53 KO	1072	1 (0.1%)	0 (0.0%)	0 (0.0%)	-3	1068 (99.6%)	-4	2 (0.2%)	0	1 (0.1%)	-60	1 (0.1%)
GEIC-Plate31-C06	MSTO-211H p53 KO	1477	466 (31.6%)	0 (0.0%)	0 (0.0%)	-2	982 (66.5%)	0	488 (33.0%)	-1	3 (0.2%)	-23	2 (0.1%)
GEIC-Plate31-C07	MSTO-211H p53 KO	1385	1355 (97.8%)	0 (0.0%)	0 (0.0%)	0	1379 (99.6%)	-1	5 (0.4%)	-36	1 (0.1%)	NA	
GEIC-Plate31-C08	MSTO-211H p53 KO	1158	1127 (97.3%)	0 (0.0%)	0 (0.0%)	0	1155 (99.7%)	-1	3 (0.3%)	NA		NA	
GEIC-Plate31-C09	MSTO-211H p53 KO	1555	1005 (64.6%)	0 (0.0%)	0 (0.0%)	0	1030 (66.2%)	-15	522 (33.6%)	-1	3 (0.2%)	NA	
GEIC-Plate31-C10	MSTO-211H p53 KO	1313	684 (52.1%)	0 (0.0%)	0 (0.0%)	0	696 (53.0%)	-2	616 (46.9%)	-3	1 (0.1%)	NA	
GEIC-Plate31-C11	MSTO-211H p53 KO	1257	2 (0.2%)	0 (0.0%)	0 (0.0%)	1	1252 (99.6%)	0	5 (0.4%)	NA		NA	
GEIC-Plate31-C12	MSTO-211H p53 KO	1125	0 (0.0%)	0 (0.0%)	0 (0.0%)	-2	1123 (99.8%)	-3	1 (0.1%)	-4	1 (0.1%)	NA	
GEIC-Plate31-D01	MSTO-211H p53 KO	1600	0 (0.0%)	0 (0.0%)	0 (0.0%)	-10	810 (50.6%)	-9	789 (49.3%)	-11	1 (0.1%)	NA	
GEIC-Plate31-D02	MSTO-211H p53 KO	1376	432 (31.4%)	0 (0.0%)	0 (0.0%)	-3	488 (35.5%)	-22	442 (32.1%)	0	438 (31.8%)	-10	3 (0.2%)
GEIC-Plate31-D03	MSTO-211H p53 KO	1399	1369 (97.9%)	0 (0.0%)	0 (0.0%)	0	1396 (99.8%)	-1	3 (0.2%)	NA		NA	
GEIC-Plate31-D04	MSTO-211H p53 KO	1331	1302 (97.8%)	0 (0.0%)	1 (0.1%)	0	1323 (99.4%)	-1	7 (0.5%)	-59	1 (0.1%)	NA	
GEIC-Plate31-D05	MSTO-211H p53 KO	555	1 (0.2%)	0 (0.0%)	0 (0.0%)	-40	552 (99.5%)	0	2 (0.4%)	-41	1 (0.2%)	NA	
GEIC-Plate31-D06	MSTO-211H p53 KO	1078	1048 (97.2%)	0 (0.0%)	0 (0.0%)	0	1069 (99.2%)	-1	4 (0.4%)	-6	3 (0.3%)	-7	1 (0.1%)
GEIC-Plate31-D07	MSTO-211H p53 KO	1565	744 (47.5%)	0 (0.0%)	0 (0.0%)	-2	792 (50.6%)	0	771 (49.3%)	-3	1 (0.1%)	-6	1 (0.1%)
GEIC-Plate31-D08	MSTO-211H p53 KO	1329	1294 (97.4%)	0 (0.0%)	0 (0.0%)	0	1323 (99.5%)	-1	4 (0.3%)	-44	2 (0.2%)	NA	
GEIC-Plate31-D09	MSTO-211H p53 KO	1445	1406 (97.3%)	0 (0.0%)	0 (0.0%)	0	1439 (99.6%)	-1	6 (0.4%)	NA		NA	
GEIC-Plate31-D10	MSTO-211H p53 KO	1129	1095 (97.0%)	0 (0.0%)	0 (0.0%)	0	1125 (99.6%)	-1	3 (0.3%)	-2	1 (0.1%)	NA	
GEIC-Plate31-D11	MSTO-211H p53 KO												
GEIC-Plate31-D12	MSTO-211H p53 KO	1267	621 (49.0%)	0 (0.0%)	0 (0.0%)	0	630 (49.7%)	-18	319 (25.2%)	-28	313 (24.7%)	-1	2 (0.2%)

Supplemental Table S4: JK181_hTP53.ex4 summary.xlsx

Name	Sample	Total	guide4	#1-Indel	#1-Reads(%)	#2-Indel	#2-Reads(%)	#3-Indel	#3-Reads(%)	#4-Indel	#4-Reads(%)
GEIC-Plate30-A01	MSTO-211H p53 KO	978	958 (98.0%)	0	977 (99.9%)	-1	1 (0.1%)	NA		NA	
GEIC-Plate30-A02	MSTO-211H p53 KO	483	88 (18.2%)	-14	391 (81.0%)	0	89 (18.4%)	-15	3 (0.6%)	NA	
GEIC-Plate30-A03	MSTO-211H p53 KO	145	142 (97.9%)	0	144 (99.3%)	-2	1 (0.7%)	NA		NA	
GEIC-Plate30-A04	MSTO-211H p53 KO	1037	133 (12.8%)	-1	883 (85.1%)	0	135 (13.0%)	-2	9 (0.9%)	1	5 (0.5%)
GEIC-Plate30-A05	MSTO-211H p53 KO	413	405 (98.1%)	0	411 (99.5%)	-1	1 (0.2%)	1	1 (0.2%)	NA	
GEIC-Plate30-A06	MSTO-211H p53 KO	194	171 (88.1%)	0	173 (89.2%)	-18	15 (7.7%)	-1	3 (1.5%)	6	3 (1.5%)
GEIC-Plate30-A07	MSTO-211H p53 KO	340	336 (98.8%)	0	337 (99.1%)	-1	3 (0.9%)	NA		NA	
GEIC-Plate30-A08	MSTO-211H p53 KO	495	488 (98.6%)	0	495 (100.0%)	NA		NA		NA	
GEIC-Plate30-A09	MSTO-211H p53 KO	100	92 (92.0%)	0	99 (99.0%)	-1	1 (1.0%)	NA		NA	
GEIC-Plate30-A10	MSTO-211H p53 KO	433	117 (27.0%)	2	308 (71.1%)	0	123 (28.4%)	3	1 (0.2%)	1	1 (0.2%)
GEIC-Plate30-A11	MSTO-211H p53 KO	184	180 (97.8%)	0	181 (98.4%)	-1	3 (1.6%)	NA		NA	
GEIC-Plate30-A12	MSTO-211H p53 KO	272	38 (14.0%)	10	230 (84.6%)	0	38 (14.0%)	9	3 (1.1%)	-15	1 (0.4%)
GEIC-Plate30-B01	MSTO-211H p53 KO	1023	738 (72.1%)	0	750 (73.3%)	-6	269 (26.3%)	-1	3 (0.3%)	-7	1 (0.1%)
GEIC-Plate30-B02	MSTO-211H p53 KO	1276	1259 (98.7%)	0	1270 (99.5%)	-1	6 (0.5%)	NA		NA	
GEIC-Plate30-B03	MSTO-211H p53 KO	694	49 (7.1%)	-2	641 (92.4%)	0	51 (7.3%)	-3	2 (0.3%)	NA	
GEIC-Plate30-B04	MSTO-211H p53 KO	834	532 (63.8%)	0	542 (65.0%)	-22	290 (34.8%)	-23	2 (0.2%)	NA	
GEIC-Plate30-B05	MSTO-211H p53 KO	754	738 (97.9%)	0	752 (99.7%)	-1	2 (0.3%)	NA		NA	
GEIC-Plate30-B06	MSTO-211H p53 KO	885	60 (6.8%)	-2	820 (92.7%)	0	60 (6.8%)	-3	3 (0.3%)	-1	1 (0.1%)
GEIC-Plate30-B07	MSTO-211H p53 KO	1198	733 (61.2%)	0	745 (62.2%)	-24	249 (20.8%)	-31	105 (8.8%)	-4	95 (7.9%)
GEIC-Plate30-B08	MSTO-211H p53 KO	722	704 (97.5%)	0	722 (100.0%)	NA		NA		NA	
GEIC-Plate30-B09	MSTO-211H p53 KO	636	38 (6.0%)	-25	309 (48.6%)	1	288 (45.3%)	0	39 (6.1%)	NA	
GEIC-Plate30-B10	MSTO-211H p53 KO	873	858 (98.3%)	0	867 (99.3%)	-1	6 (0.7%)	NA		NA	
GEIC-Plate30-B11	MSTO-211H p53 KO	1122	217 (19.3%)	-2	899 (80.1%)	0	219 (19.5%)	-3	3 (0.3%)	-1	1 (0.1%)
GEIC-Plate30-B12	MSTO-211H p53 KO	195	180 (92.3%)	0	180 (92.3%)	-2	10 (5.1%)	-1	5 (2.6%)	NA	
GEIC-Plate30-C01	MSTO-211H p53 KO	280	103 (36.8%)	-6	172 (61.4%)	0	106 (37.9%)	-5	1 (0.4%)	-1	1 (0.4%)
GEIC-Plate30-C02	MSTO-211H p53 KO	170	169 (99.4%)	0	169 (99.4%)	-1	1 (0.6%)	NA		NA	
GEIC-Plate30-C03	MSTO-211H p53 KO	299	293 (98.0%)	0	297 (99.3%)	-1	2 (0.7%)	NA		NA	
GEIC-Plate30-C04	MSTO-211H p53 KO										
GEIC-Plate30-C05	MSTO-211H p53 KO	1096	0 (0.0%)	1	1088 (99.3%)	0	7 (0.6%)	-1	1 (0.1%)	NA	
GEIC-Plate30-C06	MSTO-211H p53 KO										
GEIC-Plate30-C07	MSTO-211H p53 KO										
GEIC-Plate30-C08	MSTO-211H p53 KO										
GEIC-Plate30-C09	MSTO-211H p53 KO										
GEIC-Plate30-C10	MSTO-211H p53 KO	1131	1116 (98.7%)	0	1129 (99.8%)	-1	2 (0.2%)	NA		NA	
GEIC-Plate30-C11	MSTO-211H p53 KO	1766	1733 (98.1%)	0	1762 (99.8%)	-1	3 (0.2%)	-2	1 (0.1%)	NA	
GEIC-Plate30-C12	MSTO-211H p53 KO										
GEIC-Plate30-D01	MSTO-211H p53 KO										
GEIC-Plate30-D02	MSTO-211H p53 KO	1361	2 (0.1%)	-30	1351 (99.3%)	-31	8 (0.6%)	0	2 (0.1%)	NA	
GEIC-Plate30-D03	MSTO-211H p53 KO	1448	0 (0.0%)	-28	1441 (99.5%)	-29	6 (0.4%)	-6	1 (0.1%)	NA	
GEIC-Plate30-D04	MSTO-211H p53 KO										
GEIC-Plate30-D05	MSTO-211H p53 KO										
GEIC-Plate30-D06	MSTO-211H p53 KO	602	10 (1.7%)	-23	592 (98.3%)	0	10 (1.7%)	NA		NA	
GEIC-Plate30-D07	MSTO-211H p53 KO										
GEIC-Plate30-D08	MSTO-211H p53 KO										
GEIC-Plate30-D09	MSTO-211H p53 KO	1237	5 (0.4%)	-24	615 (49.7%)	-31	614 (49.6%)	0	5 (0.4%)	-30	1 (0.1%)
GEIC-Plate30-D10	MSTO-211H p53 KO	1630	1131 (69.4%)	0	1141 (70.0%)	-6	483 (29.6%)	-1	6 (0.4%)	NA	
GEIC-Plate30-D11	MSTO-211H p53 KO	1554	7 (0.5%)	-6	793 (51.0%)	-1	752 (48.4%)	0	7 (0.5%)	-2	2 (0.1%)
GEIC-Plate30-D12	MSTO-211H p53 KO										
